# Inverse‐Electron‐Demand Diels–Alder Reaction of Tropone with Graphene Supported on Cu(111)

**DOI:** 10.1002/smll.202503669

**Published:** 2025-09-30

**Authors:** Jia Tu, Wentong Zhou, Lawrence M. Wolf, Mingdi Yan

**Affiliations:** ^1^ Department of Chemistry University of Massachusetts Lowell Lowell MA 01854 USA

**Keywords:** Cu(111), DFT calculations, Diels–Alder reaction, graphene, tropone

## Abstract

An inverse‐electron‐demand Diels–Alder (IEDDA) reaction between graphene supported on Cu(111) and tropone catalyzed by a Lewis acid is reported. Reaction catalyzed by B(C_6_F_5_)_3_ resulted in a significant change in the Raman G band and the appearance of carbonyl group in the functionalized graphene. Treating the product with a reducing agent NaBH_4_ or hydrazine led to a near complete disappearance of shoulder peaks in the Raman G band and a decrease in carbonyl intensity. On the other hand, reaction catalyzed by BPh_3_ resulted in the appearance of C─O group in the functionalized graphene. The findings support [4 + 2] and [8 + 2] cycloaddition for the B(C_6_F_5_)_3_‐ and BPh_3_‐catalyzed reaction, respectively. Density functional theory (DFT) calculations revealed that favorable cycloaddition reactions with tropone can be achieved through the usage of curved graphene. The origin of the Lewis acid‐dependent selectivity inversion is predicted to be based on the requirement for Lewis acid dissociation during the course of the [8 + 2] reaction with B(C_6_F_5_)_3_ dissociation requiring more energy than BPh_3_ dissociation. This study represents a new strategy in graphene chemistry that combines synergistic activation of graphene via substrate interactions and of tropone via Lewis acid coordination, as well as catalyst selection to modulate reaction pathways.

## Graphical abstract

The inverse‐electron‐demand Diels–Alder reaction between tropone and graphene supported on Cu(111), catalyzed by B(C_6_F_5_)_3_ or BPh_3_, gave [4 + 2] and [8 + 2] cycloadducts, respectively. Computations reveal that the cycloadditions become favorable on curved graphene with secondary substrate charge transfer effects and reveal the origins of the product selectivity reversal to be based on product Lewis acid coordination strength.

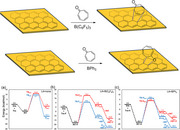

## 1 Introduction

Chemical functionalization of graphene is a powerful strategy for tailoring its chemical and electronic properties, enabling diverse applications in electronics, energy, catalysis, and biomedicine.^[^ ^1^ ^]^ However, the range of chemical reactions applicable to graphene remains limited due to its extended π‐conjugation which significantly lowers its chemical reactivity. Reported methods include radical reactions with diazonium ions or halogens, [2 + 1] cycloadditions with nitrenes, carbenes or malonate derivatives, 1,3‐dipolar cycloaddition with azomethine ylides, [2 + 2] cycloadditions with arynes, and [4 + 2] cycloadditions.^[^ ^1^, ^2^, ^3^, ^4^, ^5^, ^6^, ^7^ ^]^

The Diels–Alder (DA) reaction is a [4 + 2] cycloaddition between a conjugated diene and an alkene (dienophile) to form a six‐membered ring. The DA reaction is known for its ability to form new C─C bonds, atom efficiency, broad functional group compatibility, and mild reaction conditions. In principle, graphene can function as either a diene or a dienophile in Diels–Alder reactions.^[^ ^3^, ^5^, ^8^ ^]^ Table S1 (Supporting Information) summarized the DA reactions on graphene reported in the literature. Additionally, some Diels–Alder reactions are reversible, which can reversibly tune the electronic and photonic properties of graphene in a straightforward way under mild conditions without the formation of residual conjugated π‐radicals or by‐products.^[^ ^4^, ^5^ ^]^ However, for pristine graphene, computational results revealed that the energy is too high for the DA reactions to occur on the graphene basal plane (Table S2, Supporting Information). As such, it was proposed that the reactions occurred on the defect sites and edges on graphene instead.^[^ ^9^, ^10^ ^]^ Subsequently, the DA reactions on graphene have been carried out by activating graphene, for example, through strains or charge doping. For instance, Braunschweig and coworkers employed force on graphene to accelerate the DA reaction.^[^ ^11^ ^]^ Altenburg et al. used Ir(111) as the substrate to form a moiré pattern on graphene to selectively functionalize graphene with iron phthalocyanines via a non‐classic Diels–Alder reaction.^[^ ^12^ ^]^ Li et al. found that *cis*‐diene with two dihydronaphthalene molecules enabled DA reactions with graphene.^[^ ^13^ ^]^

We found that metals, such as Ni and Cu, as the substrate enhanced the reactivity of graphene in the [2 + 1] cycloadditions with nitrenes and in the DA reactions with electron‐rich diene 2,3‐dimethoxybutadiene (DMBD) and electron‐deficient dienophile maleic anhydride (MA).^[^ ^10^ ^]^ Computations revealed that the metal substrate lowers the activation energy by facilitating charge transfer interactions with graphene. Additionally, computation revealed a lower activation energy, but the product from DMBD to be more stable. Based on these results, we hypothesize that since the graphene supported on a metal is electron‐rich, and a diene reactant leads to a more stable product, that the [4 + 2] reaction on graphene may inherently favor an inverse‐electron‐demand Diels–Alder (IEDDA) reaction where an electron‐deficient diene would react more favorably with substrate‐supported graphene. In this work, we carried out the IEDDA reactions of graphene supported on Cu(111) with an electron‐deficient diene. Cu(111) was selected as the substrate for several reasons. 1) Cu(111) is among the best substrates for the fabrication of high‐quality CVD graphene due to its low carbon solubility, well‐matched symmetry, and lattice constants that promote large area growth of uniform monolayer graphene.^[^ ^14^, ^15^, ^16^ ^]^ 2) Cu(111) can cause n‐type doping in graphene.^[^ ^17^, ^18^, ^19^, ^20^ ^]^ This is a result of the work function difference between copper (5.22 eV) and graphene (4.48 eV for freestanding graphene).^[^ ^17^, ^18^, ^19^, ^20^, ^21^, ^22^ ^]^ Inverse photoelectron spectroscopy indicated a substrate‐to‐graphene charge transfer of ≈‐0.03 electrons per carbon atom.^[^ ^18^ ^]^ 3) Graphene on Cu(111) experiences uniform biaxial compression of ≈0.3% strain, arising from difference in thermal expansion coefficients between graphene and Cu, where cooling after growth leads to a contraction of the Cu lattice and an expansion of the graphene lattice.^[^ ^21^ ^]^ The strain on graphene also can enhance its chemical reactivity.^[^ ^23^, ^24^ ^]^ 4) The equilibrium separation between graphene and Cu (111) surface is ≈3.3 Å, indicating weak interaction with graphene through physisorption, which does not destroy the electronic structure of graphene.^[^ ^23^, ^24^ ^]^ Taken together, the ease of fabrication, preservation of graphene electronic structure combined with n‐doping and strain effect makes graphene on Cu(111) an ideal system to test the reactivity of graphene as an electron‐rich dienophile.

**Scheme 1:**
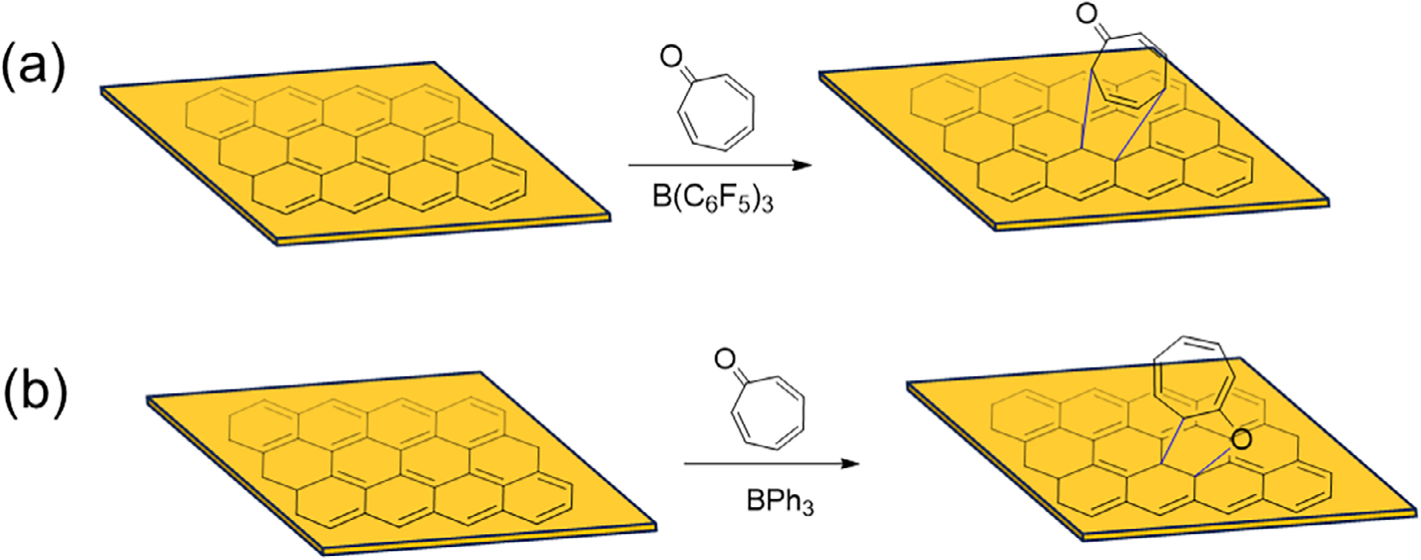
Cycloaddition reaction of graphene with tropone via a) [4 + 2] mechanism catalyzed by B(C_6_F_5_)_3_, and b) [8 + 2] mechanism catalyzed by BPh_3_.

Tropone was selected as a model diene in this work (**Scheme** **1**). Tropone is an electron‐deficient diene as the carbonyl is polarized toward the electronegative oxygen.^[^ ^25^, ^26^, ^27^ ^]^ It can undergo IEDDA reactions with a variety of electron‐rich dienophiles at high temperature or high pressures characteristic of unsaturated dienes.^[^ ^28^, ^29^, ^30^, ^31^, ^32^ ^]^ It has been shown that Lewis acids enhanced the electron deficiency of tropone and enabled IEDDA reactions at room temperature.^[^ ^26^, ^33^ ^]^ We therefore investigated the Lewis acid‐catalyzed IEDDA reactions of graphene supported on Cu(111) with tropone. We found that when using tris(pentafluorophenyl)borane, B(C_6_F_5_)_3_, as the catalyst, the reaction proceeded at room temperature with or without a solvent. The effects of reaction time, temperature, solvent, substrate, and Lewis acid were investigated. Raman spectroscopy revealed significant changes in the graphene G band after the reaction, showing shoulder peaks resulting from oxygen‐containing species. X‐ray photoelectron spectroscopy (XPS) confirmed the presence of carbonyl groups in the product. Treating the product with a reducing agent, NaBH_4_ or hydrazine, led to a near complete disappearance of the Raman shoulder peaks. Interestingly, using BPh_3_ as the Lewis acid catalyst gave no shoulder peaks in the Raman G band, however, the Raman D band appeared and XPS detected C─O groups in the product. This is in contrast with the reaction catalyzed by B(C_6_F_5_)_3_, which produced C═O rather than C─O structures. Based on these findings, we hypothesize a [4 + 2] cycloaddition mechanism for the B(C_6_F_5_)_3_‐catalyzed reaction (Scheme 1a) and a [8 + 2] cycloaddition mechanism for the BPh_3_‐catalyzed reaction (Scheme 1b).

## 2 Results and Discussion

### 2.1 IEDDA Reaction of Tropone with Graphene Supported on Cu(111) Catalyzed by Lewis Acid B(C_6_F_5_)_3_

Monolayer graphene on Cu(111) (Gra/Cu(111)) was prepared using a home‐built chemical vapor deposition (CVD) apparatus by first annealing polycrystalline Cu foil at 1060 °C for 3 h, and then growing monolayer graphene from methane gas at 1060 °C for 30 min.^[^ ^34^ ^]^ Raman spectra of the prepared graphene showed either no or minimal D peak, with *A_D_*/*A_G_* of 0.033 ± 0.015 and *A_2D_*/*A_G_* of 2.8 ± 0.66, respectively (Figure 3a; Figure S1f, Supporting Information), consistent with high‐quality monolayer graphene.

**Figure 1:**
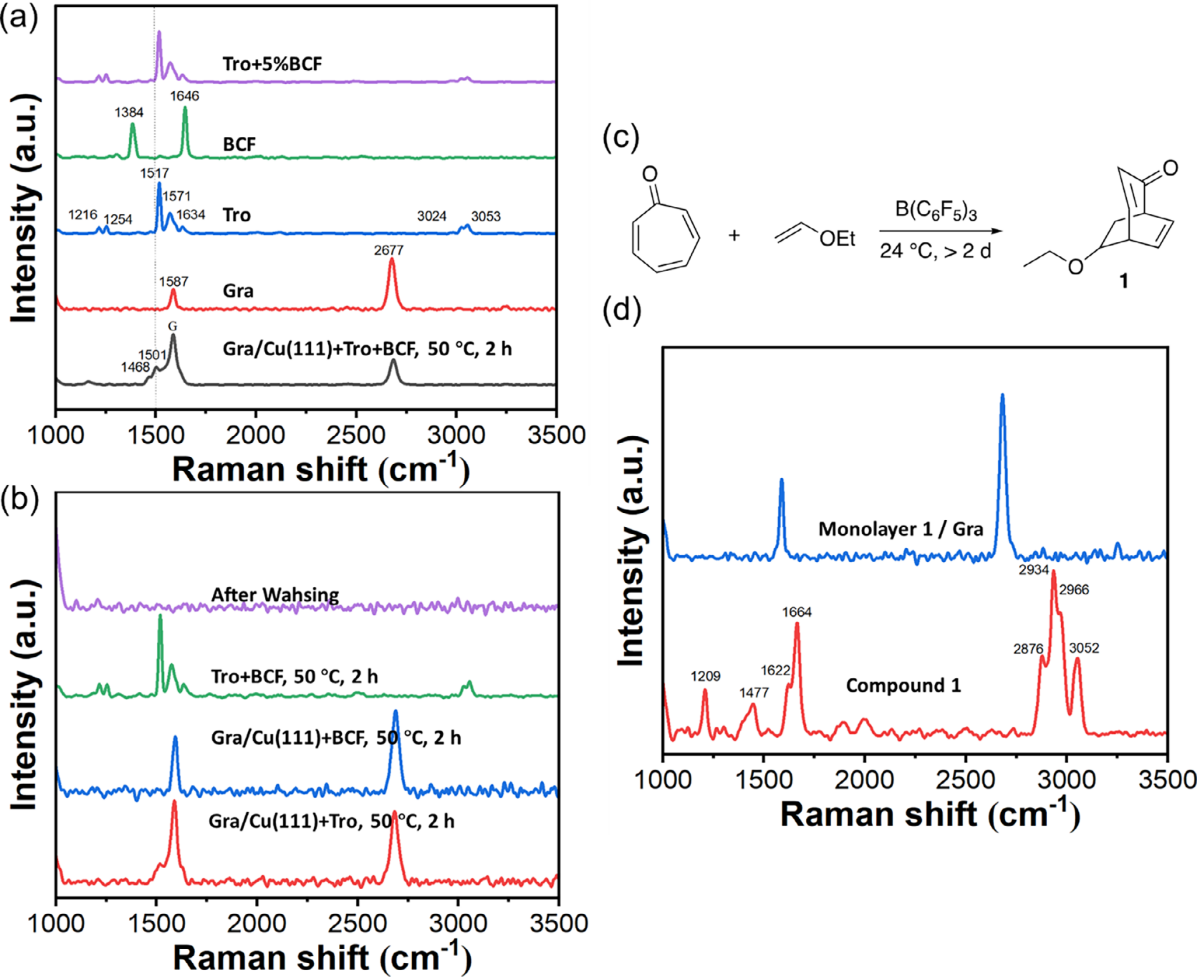
a) Raman spectra of tropone‐functionalized graphene, graphene (Gra), tropone (Tro), B(C_6_F_5_)_3_ (BCF), and Tro + 5%BCF. b) Raman spectra of tropone‐functionalized graphene (grey) and control samples, including Gra/Cu(111) treated with tropone only at 50 °C for 2 h (red), Gra/Cu(111) treated with B(C_6_F_5_)_3_ only at 50 °C for 2 h (blue), Tro + 5% BCF deposited on silicon wafer and heated at 50 °C for 2 h before (green) and after (purple) washing with toluene, tetrahydrofuran, and acetone. Additional spectra of each sample can be found in Figure S1 (Supporting Information). c) [4 + 2] IEDDA reaction of tropone with ethyl vinyl ether to give compound **1**. d) Raman spectra of compound **1** (red), and monolayer of compound **1** on graphene (blue). Additional Raman spectra of each sample and the sample preparation procedure for monolayer compound **1** on graphene can be found in Figure S3 (Supporting Information).

Initially, the reaction was carried out under solvent‐free conditions, where tropone mixed with 5 mol% B(C_6_F_5_)_3_ was placed on graphene and was heated at 50 °C for 2 h. After extensive washing with solvents (twice with toluene, twice with tetrahydrofuran (THF), and thrice with acetone), the samples were characterized by Raman spectroscopy. Compared to graphene having a symmetrical G band at 1587 cm^−1^ (**Figure** **1**a, red curve), the G band in tropone‐functionalized graphene broadened, and two new shoulder peaks at 1501 and 1468 cm^−1^ appeared (Figure 1a, grey curve). These peaks are not from tropone (Figure 1a, blue curve) or B(C_6_F_5_)_3_ (Figure 1a, green curve) based on comparison of their Raman spectra. The Raman spectrum of tropone contains similar characteristic peaks as its infrared (IR) spectrum (Figure S2c, Supporting Information, main Raman and IR bands of tropone are listed in Table S3, Supporting Information): aromatic C─H stretching at 3024 and 3053 cm^−1^, C═O stretching at 1634 cm^−1^, C═C stretching at 1517 and 1571 cm^−1^, and C─H bending vibrations at 1216 and 1254 cm^−1^.^[^ ^35^ ^]^ The Raman spectrum of B(C_6_F_5_)_3_ contains two main peaks: aromatic C═C stretching at 1646 cm^−1^ and C─F stretching at 1384 cm^−1^.^[^ ^36^ ^]^ The two new shoulder peaks at 1501 or 1468 cm^−1^ in tropone‐functionalized graphene do not match those of tropone or B(C_6_F_5_)_3_.

Three additional experiments were conducted to further confirm the functionalization of graphene by tropone. In the first experiment, several control samples were prepared, including Gra/Cu(111) treated with tropone without B(C_6_F_5_)_3_, Gra/Cu(111) treated with B(C_6_F_5_)_3_ without tropone, and tropone + 5% B(C_6_F_5_)_3_ on silicon wafer without graphene. All samples were heated at 50 °C for 2 h. For Gra/Cu(111) treated with tropone alone, the shoulder peaks in the Raman G band were also observed (Figure 1b, red curve), but the peak intensities were lower than Gra/Cu(111) treated with tropone in the presence of B(C_6_F_5_)_3_ (Figure 1a, grey curve). Gra/Cu(111) treated with B(C_6_F_5_)_3_ alone without tropone did not show any new peaks except for the G and 2D bands of graphene (Figure 1b, blue curve). Without graphene, heating the mixture of tropone and 5% B(C_6_F_5_)_3_ on silicon wafer at 50 °C for 2 h resulted in peaks corresponding to tropone due to the low concentration of B(C_6_F_5_)_3_ (Figure 1b, green curve). However, these peaks completely disappeared after washing with the solvents (Figure 1b, purple curve), indicating that the reagents were physisorbed and were removed by solvents. Taken together, the newly appeared shoulder peaks at 1501 and 1468 cm^−1^ can be attributed to the reaction of graphene with tropone. The Lewis acid, B(C_6_F_5_)_3,_ facilitated the reaction of Gra/Cu(111) with tropone, judging by the higher intensities of these shoulder peaks. The IEDDA reaction did proceed without B(C_6_F_5_)_3_, but to a much lesser degree.

In the second experiment, a model reaction was carried out between tropone and ethyl vinyl ether (Figure 1c). The product, compound **1**, was characterized by proton nuclear magnetic resonance (^1^H NMR) and IR spectroscopy (Figure S2, Supporting Information). Raman and IR absorption bands of compound **1** and tropone are listed in Table S3 (Supporting Information). The major Raman bands in compound **1**, such as the strong C─H stretching in the range of 2876–3052 cm^−1^, and the C═O and C═C stretching at 1666 and 1634 cm^−1^ (Figure 1d, red curve), were absent in the tropone‐functionalized graphene (Figure 1a, grey curve). One possibility is that the concentration of the reaction product is too low, as a maximum of one product layer is formed on graphene after the reaction. To test this, a sample was prepared by depositing an equivalent of a monolayer of compound **1** on graphene (see protocol in Figure S3, Supporting Information caption). In the Raman spectrum, the Raman bands belonging to compound **1** was not detected and only the graphene G and 2D bands were seen (Figure 1d, blue curve). This result demonstrates that even when the reaction occurs, the product may not be detectable by Raman due to low concentration.

The third experiment tested reversibility of the product, as Diels–Alder reactions on graphene can be reversible (see examples in Table S1, Supporting Information). The cycloreversion reactions of Diels–Alder graphene adducts generally occur at high temperatures (>100 °C).^[^ ^9^ ^]^ For example, in the cycloreversion of tropone‐functionalized C_60_, the reaction was carried out at 160 °C.^[^ ^37^ ^]^ To test the possibility of cycloreversion, tropone‐functionalized graphene was heated in toluene at 100 °C for 4.5 or 19 h, or in 1,2‐dichlorobenzene at 160 °C for 19 h (Figure S4a, Supporting Information). Raman spectra of all three samples showed the shoulder peaks of the G band like the tropone‐functionalized graphene (Figure S4b,c, Supporting Information). Heating unfunctionalized pristine graphene in toluene at 100 °C for 19 h did not affect the structure of graphene as the Raman spectra remained unchanged (Figure S4d, Supporting Information). These results indicate that tropone‐functionalized graphene does not undergo cycloreversion upon heating.

## 3 Analysis of Raman G Band

**Figure 2:**
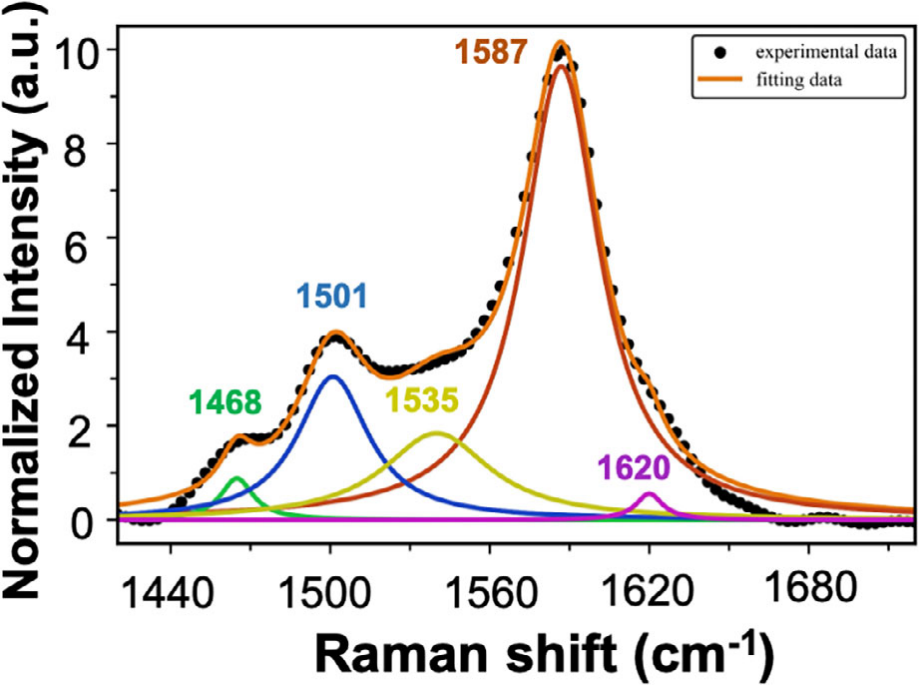
Experimental data and deconvoluted fitting of the Raman G band in tropone‐functionalized graphene. The black dots are the experimental data, and the solid orange line is the fitting curve.

From the experiments and analysis above, it can be concluded that the two new shoulder peaks in the Raman G band of tropone‐functionalized graphene resulted from the modification of graphene itself. Subsequently, the G band of the tropone‐functionalized graphene was deconvoluted in the region of 1420–1720 cm^−1^, from which five distinct peaks were obtained: 1468, 1501, 1535, 1587, and 1620 cm^−1^ (**Figure** **2**). The intensities of peaks at 1501 and 1535 cm^−1^ were much higher than those at 1468 and 1620 cm^−1^. The fitting (orange line) aligns well with the experimental data (black dots).

The peak at 1587 cm^−1^ is the intrinsic G band of graphene, which is the first‐order Raman mode originated from the in‐plane stretching of *sp^2^* carbon.^[^ ^38^ ^]^ The peak at 1620 cm^−1^, also known as the D' band, is associated with intravalley scattering and is activated by defects within the graphene lattice.^[^ ^38^ ^]^ This peak can be created by, for example, vacancies or atomic displacements in graphene by ion irradiation, localized defects or disorder by plasma etching carbon atoms, bond length variations or local disorder by mechanical stress or strain.^[^ ^39^, ^40^, ^41^, ^42^ ^]^ Peaks at 1501 and 1535 cm^−1^ are typically associated with the D″ band, which is associated with amorphous carbon phases, interstitial defects, or disorder in the *sp^2^* network.^[^ ^43^, ^44^, ^45^ ^]^ One main contribution to the appearance of the D″ peak in pristine graphene is oxidation. The introduction of oxygen‐containing groups, including hydroxyl (─OH), epoxy (─O─), carbonyl (─C═O), and carboxyl (─COOH) groups, disrupts the *sp^2^* network, causing additional phonon modes, including the D″ band.^[^ ^46^, ^47^ ^]^ For instance, graphene oxide (GO) and reduced GO (rGO) with high carboxyl content often exhibit a broad D″ peak near 1535 cm^−1^.^[^ ^46^, ^47^, ^48^ ^]^ The appearance of the 1468 cm^−1^ peak in pristine graphene has also been attributed to oxidation associated with disordered *sp^2^*/*sp^3^* hybridized structures. For instance, GO and rGO often exhibit broad peaks in the 1400–1500 cm^−1^ range due to mixed *sp^2^* and *sp^3^* bonding.^[^ ^47^ ^]^

Interestingly, the Raman of tropone‐functionalized graphene did not show obvious D band. In graphene and related carbon materials, the D band (≈1350 cm^−1^) and the D′ band (≈1620 cm^−1^) are both defect‐activated Raman modes, however, they originate from different scattering mechanisms and have distinct physical implications.^[^ ^38^, ^41^ ^]^ The D band arises from intervalley scattering, where the electron is scattered between two different valleys (K and K′) in the Brillouin zone, assisted by a phonon and a defect. The D′ band originates from intravalley scattering, where the electron is scattered within the same valley (K → K or K′ → K′) with the assistance of a phonon and a defect. The D band is activated by vacancies, grain boundaries, edges, or functional groups in the graphene lattice.^[^ ^38^ ^]^ It is not present in defect‐free graphene but appears strongly in GO, rGO, or mechanically damaged graphene. The D′ band is activated by point defects, such as oxygen functionalization, doping, or small lattice distortions, and appears in doped or chemically functionalized graphene, such as oxidized or hydrogenated graphene.^[^ ^40^, ^46^ ^]^ Generally, *I_D_*/*I_G_* measures defect density, *I_D’_*/*I_G_* correlates with doping/oxidation level.^[^ ^49^, ^50^ ^]^

The presence of a D′ peak (≈1620 cm^−1^) without a D peak (≈1350 cm^−1^) in the Raman spectrum of tropone‐functionalized graphene may indicate specific types of disorder or functionalization mechanism. Possibilities include: 1) Low level of disorder, where only certain types of defects or modifications are present. In this case, graphene lattice remains relatively intact, whereas there are minimal lattice disruptions like grain boundaries or large vacancies. 2) Doping or oxidation at specific sites. These may include substitutional doping, mild chemical functionalization, and intravalley scattering without breaking symmetry or creating extended disorder. 3) Charge transfer from functionalization or substrate interaction. This modifies electronic properties, selectively activating the D′ mode without disrupting the long‐range order in graphene. Presently, there are no analytical techniques that can determine the precise atomic level structure of functionalized graphene.

### 3.1 Effect of Temperature and Time

**Figure 3:**
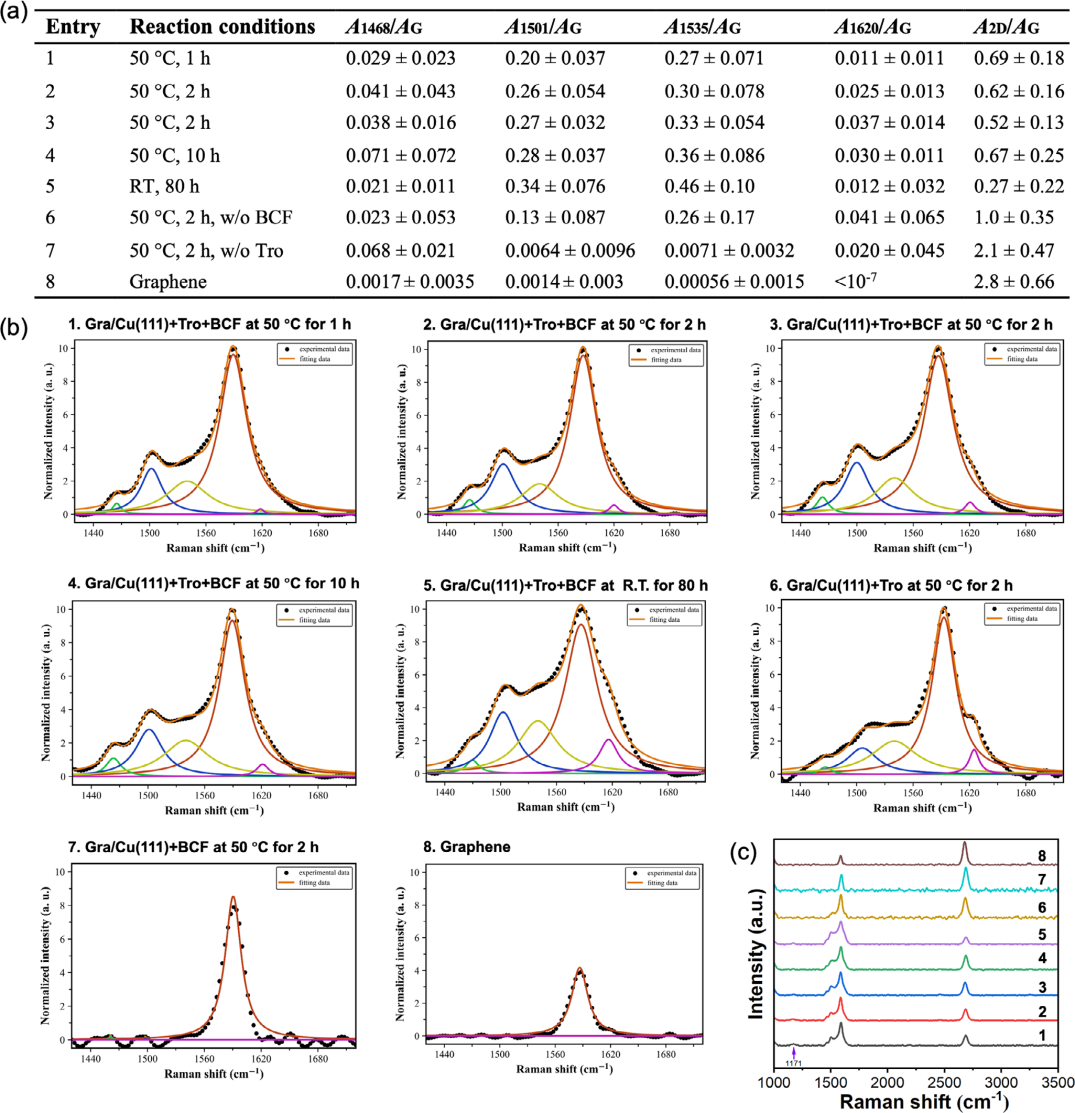
Effect of temperature and time on the reaction of Gra/Cu(111) with tropone. a) Samples, reaction conditions, and Raman data summary. Note that entry 3 is the repeat of entry 2. RT: room temperature. b) Raman G bands and deconvolution of entries 1–8. c) Typical Raman spectra of entries 1–8. All Raman spectra and graphs of *A*_1468_/*A*_G_, *A*_1501_/*A*_G_, *A*_1535_/*A*_G_, *A*_1620_/*A*_G_, and *A*_2D_/*A*_G_ can be found in Figure S5 (Supporting Information).

The effect of temperature and time on the IEDDA reaction of Gra/Cu(111) with tropone using Lewis acid B(C_6_F_5_)_3_ was explored (**Figure** **3**a), including, 50 °C for 1, 2, and 10 h (entries 1‐4), and room temperature for 80 h (entry 5). The reaction of Gra/Cu(111) with tropone without B(C_6_F_5_)_3_ (entry 6), treating Gra/Cu(111) with B(C_6_F_5_)_3_ without tropone (entry 7), and unfunctionalized pristine graphene (entry 8) were included for comparison. Raman spectra of each sample were recorded (Figure 3c) and the area ratio of the deconvoluted peak versus the G peak at 1587 cm^−1^ as well as the area ratio of 2D peak versus the G peak, *A*_2D_/*A*_G_ were calculated (Figure 3a). The 2D versus G peak ratio is a parameter frequently used to analyze graphene structure, functionalization, and doping, providing insights into the number of layers, chemical modification, and electronic interactions in graphene.^[^ ^39^, ^40^, ^49^, ^51^ ^]^ The G peak represents the in‐plane vibration of *sp^2^* carbon atoms and is sensitive to doping and strain. The 2D peak, at ≈2700 cm^−1^, originates from a double‐resonance process, and is highly sensitive to changes in electronic structure, doping, defects, and strain caused by functionalization. For pristine monolayer graphene, *A*_2D_/*A*_G_ is greater than 2 (*cf*. entries 8). Functionalization of graphene disrupts the electronic structure and reduces mobility, which significantly alters the 2D band, leading to decrease in peak intensity and *A*_2D_/*A*_G_ smaller than 2. As seen in Figure 3a, *A*_2D_/*A*_G_ of tropone‐functionalized graphene were significantly lower. The most significant changes were observed in the peaks at 1501 and 1535 cm^−1^, associated with the D″ band, where the peak intensities increased significantly after the reaction. In contrast, the changes at 1468 and 1620 cm^−1^ were much smaller and had large errors.

At 50 °C, *A*_1501_/*A*_G_ and *A*_1535_/*A*_G_ of the product increased as the reaction time increased from 1 h to 2 h to 10 h, whereas *A*_2D_/*A*_G_ was similar and was in the range of 0.52–0.69 (entries 1–4). At room temperature for 80 h, *A*_1501_/*A*_G_ and *A*_1535_/*A*_G_ increased, and *A*_2D_/*A*_G_ decreased significantly to 0.27 (entry 5). This is consistent with the largest *A*_1501_/*A*_G_ (0.34) and *A*_1535_/*A*_G_ (0.46) observed, indicating high degree of functionalization under this reaction condition. Without B(C_6_F_5_)_3_ (entry 6), *A*_1501_/*A*_G_ was smaller, and *A*_2D_/*A*_G_ was larger than with B(C_6_F_5_)_3_. This result showed that without the Lewis acid catalyst, the extent of functionalization was lower than with the catalyst. Without tropone (entry 7), there was minimal change in the G peak compared to pristine graphene (entry 8), and *A*_2D_/*A*_G_ was greater than 2.

### 3.2 Reaction with Tropone or 2‐Chlorotropone in Toluene

**Figure 4:**
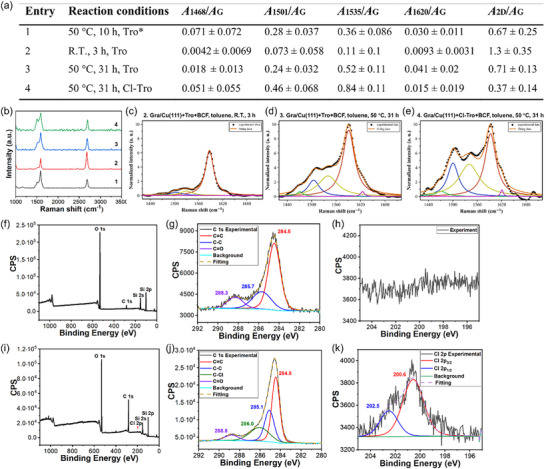
Reaction of Gra/Cu(111) with tropone or 2‐chlorotropone (Cl‐Tro) in toluene. a) Samples, reaction conditions, and Raman data summary. *: Carried out without toluene under neat condition. b) Typical Raman spectra of all samples. All Raman spectra are presented in Figure S7 (Supporting Information). c–e) Raman G band deconvolution of entries 2‐4. f) XPS survey scan, g) high‐resolution C 1s spectrum and peak deconvolution, and h) Cl 2p region of entry 3. i) XPS survey scan, j) high‐resolution C 1s spectrum and peak deconvolution, k) high‐resolution Cl 2p spectrum and peak deconvolution of entry 4. Samples were prepared on silicon wafers, and therefore the observed Si 2s and Si 2p signals in both (f,i).

The reaction was next carried out in a solvent instead of under the neat condition. Toluene was chosen due to its relatively high boiling point (110 °C), and it can dissolve both tropone and B(C_6_F_5_)_3_. In addition to tropone, 2‐chlorotropone was also tested (**Figure** **4**a). For reaction of Gra/Cu(111) with tropone at room temperature for 3 h (entry 2), a small shoulder peak was observed (Figure 4c). The intensities of deconvoluted peaks were low and *A*_2D_/*A*_G_ was 1.3 (Figure 4a). Increasing the temperature to 50 °C and reaction time to 31 h (entry 3) resulted a significant increase in the intensities of shoulder peaks in the G band (Figure 4d). Reaction with 2‐chlorotropone at 50 °C for 31 h (entry 4) gave substantial shoulder peaks (Figure 4e). Compared to the reaction with tropone under the same conditions of 50 °C for 31 h (entry 3), *A*_1501_/*A*_G_ and *A*_1535_/*A*_G_ of 2‐chlorotropone‐functionalized graphene were larger, increasing from 0.24 to 0.46 and from 0.52 to 0.84, respectively. Additionally, *A*_2D_/*A*_G_ decreased from 0.71 ± 0.13 for tropone to 0.37 ± 0.14 for 2‐chlorotropone, indicating a higher degree of structure perturbation. The higher reactivity of 2‐chlorotropone is likely due to the electron‐withdrawing Cl, which further increases the electron deficiency of 2‐chlorotropone and promotes the IEDDA reaction with the electron‐rich graphene.^[^ ^52^, ^53^, ^54^ ^]^

The reactivity of Gra/Cu(111) with tropone and 2‐chlorotropone was further studied by XPS. The XPS survey scan after rection with tropone showed the anticipated C 1s and O 1s peaks (Figure 4f). The high‐resolution C 1s spectrum revealed three peaks after deconvolution (Figure 4g). The major peak at 284.5 eV is assigned to *sp^2^* carbon characteristic of graphene.^[^ ^13^, ^55^, ^56^, ^57^, ^58^ ^]^ Peaks at 285.7 and 288.3 eV are assigned to C─C and C═O, respectively.^[^ ^55^, ^56^, ^57^, ^59^ ^]^ The C 1s peak from C─C may result from adventitious carbon in addition to the *sp^3^* carbons in the tropone‐functionalized product. No peaks belonging to B(C_6_F_5_)_3_, e.g., B 1s at ≈188.5 eV and F 1s at ≈689.4 eV,^[^ ^60^, ^61^, ^62^, ^63^ ^]^ were observed (Figure S6, Supporting Information), ruling out the possibility of signals arising from catalyst‐substrate interactions. While the Cl 2p was absent in tropone‐functionalized graphene (Figure 4h), it appeared in 2‐chlorotropone‐functionalized graphene (Figure 4i). High‐resolution Cl 2p spectrum revealed two peaks at 200.6 eV and 202.5 eV, assigned to Cl 2p_3/2_ and Cl 2p_1/2_, respectively (Figure 4k). These are characteristic of spin‐orbit splitting of Cl, and furthermore, the area ratio of Cl 2p_3/2_ and Cl 2p_1/2_ was 2:1 as expected.^[^ ^55^ ^]^ In addition, the high‐resolution C 1s spectrum contains C─Cl peak at 286.0 eV,^[^ ^55^, ^64^ ^]^ in addition to C‐C and C═O peaks at 285.1^[^ ^55^, ^59^, ^64^ ^]^ and 288.8 eV,^[^ ^55^, ^56^, ^57^ ^]^ respectively (Figure 4j). The observation of Cl 2p and C─Cl provided strong support for the successful functionalization of graphene with 2‐chlorotropone.

### 3.3 Reaction of Tropone with Graphene Supported on Silicon Wafer

**Figure 5:**
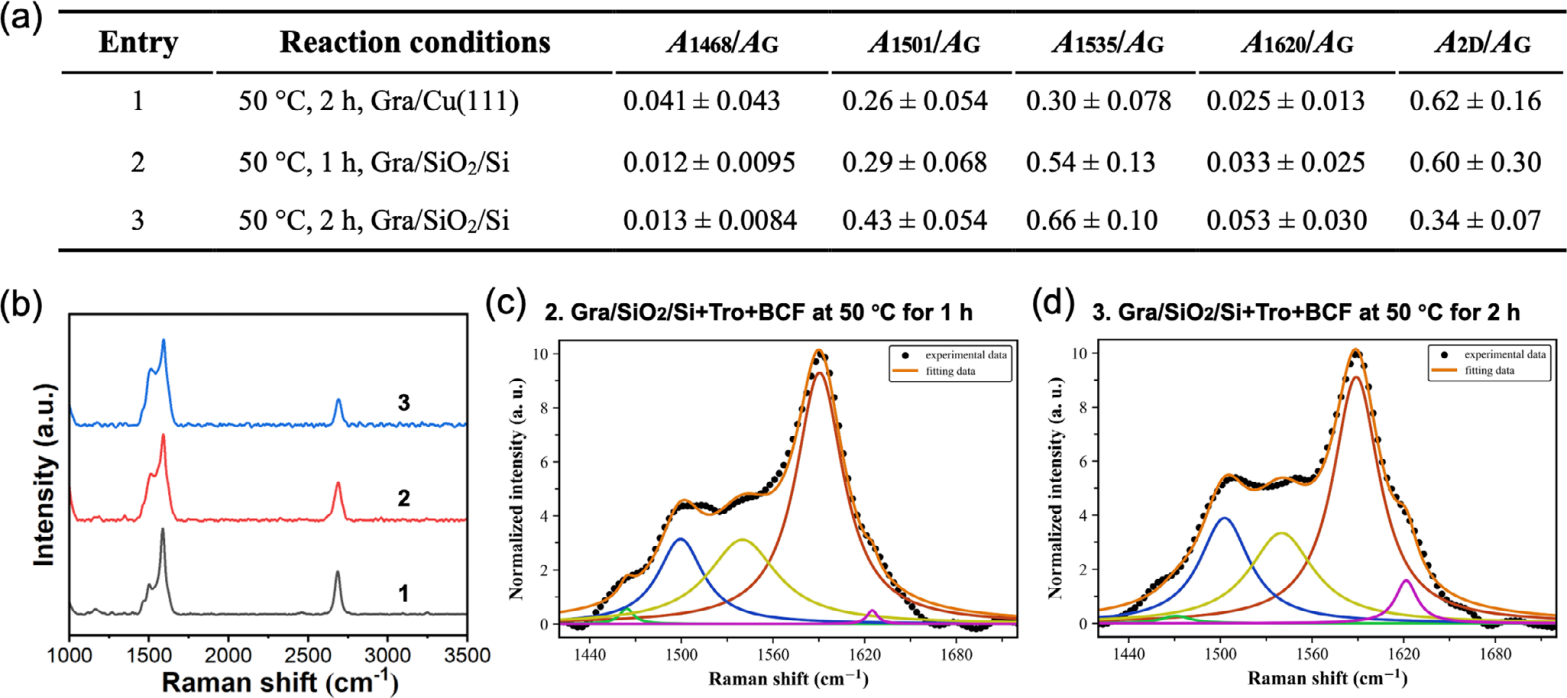
Reaction of tropone with graphene supported on silicon wafer. a) Samples, reaction conditions, and Raman data summary. All reactions were carried out without solvent. b) Typical Raman spectra of reaction products for entries 1–3. All Raman spectra for entries 2–3 are presented in Figure S8 (Supporting Information). c) G band deconvolutions of samples in entries 2 and 3. G band deconvolution of entry 1 can be found in Figure 3b.

The reaction with tropone was also conducted on graphene supported on silicon wafer (Gra/SiO_2_/Si). All reactions were carried out at 50 °C for 1 or 2 h under neat condition without a solvent (**Figure** **5**a). Similar to Gra/Cu(111), obvious shoulder peaks on the G band were observed for both Gra/SiO_2_/Si samples after functionalization (Figure 5b–d). The intensity of the shoulder peaks increased with the reaction time from 1 to 2 h, and the standard deviations of peak intensity ratios for reaction at 1 h were generally larger (Figure 5a, entry 2). Under the same reaction conditions of 50 °C for 2 h, *A*_1501_/*A*_G_ and *A*_1535_/*A*_G_ for Gra/SiO_2_/Si were higher, at 0.43 and 0.66 than Gra/Cu(111) at 0.26 and 0.30, respectively, and *A*_2D_/*A*_G_ decreased significantly from 0.62 ± 0.16 to 0.34 ± 0.07. SiO_2_/Si has been known to increase the reactivity of graphene in various reactions, attributed to the formation of electron‐hole puddles on the surface of SiO_2_/Si, which induces charge doping in graphene.^[^ ^1^, ^65^, ^66^, ^67^, ^68^ ^]^ The inhomogeneous electron‐hole puddles may also lead to large standard deviations observed in our case.

### 3.4 BPh_3_‐Catalyzed [8 + 2] Cycloaddition

**Scheme 2:**
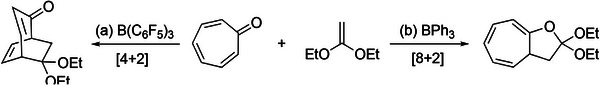
IEDDA reactions of tropone with 1,1‐diethoxyethene: a) [4 + 2] cycloaddition catalyzed by B(C_6_F_5_)_3_, b) [8 + 2] cycloaddition catalyzed by BPh_3_.^[^ ^33^ ^]^

It was reported that the IEDDA reaction of tropone gave different cycloaddition products depending on the Lewis acid used. For example, the reaction between tropone and the electron‐rich 1,1‐diethoxyethene gave the [4 + 2] cycloadduct when using B(C_6_F_5_)_3_, and [8 + 2] cycloadduct when using BPh_3_ or BF_3_·OEt_2_ (**Scheme** **2**).^[^ ^33^, ^69^, ^70^ ^]^ The Lewis acid catalyzes the reaction by lowering the LUMO of tropone, reducing the HOMO‐LUMO gap and stabilizing orbital interactions.^[^ ^70^ ^]^ The Lewis acid can also lower the activation barrier for the first bond formation.^[^ ^69^ ^]^ The regioselectivity of [4 + 2] is proposed to be originated from the rigid structure of B(C_6_F_5_)_3_ resulting in higher deformation energy and increased strain in the [8 + 2] pathway,^[^ ^70^ ^]^ or weakened nucleophilicity of the σ‐lone pair of the tropone O atom due to stronger Lewis acidity of B(C_6_F_5_)_3_,^[^ ^71^ ^]^ suppressing the [8 + 2] pathway.^[^ ^69^ ^]^

**Figure 6:**
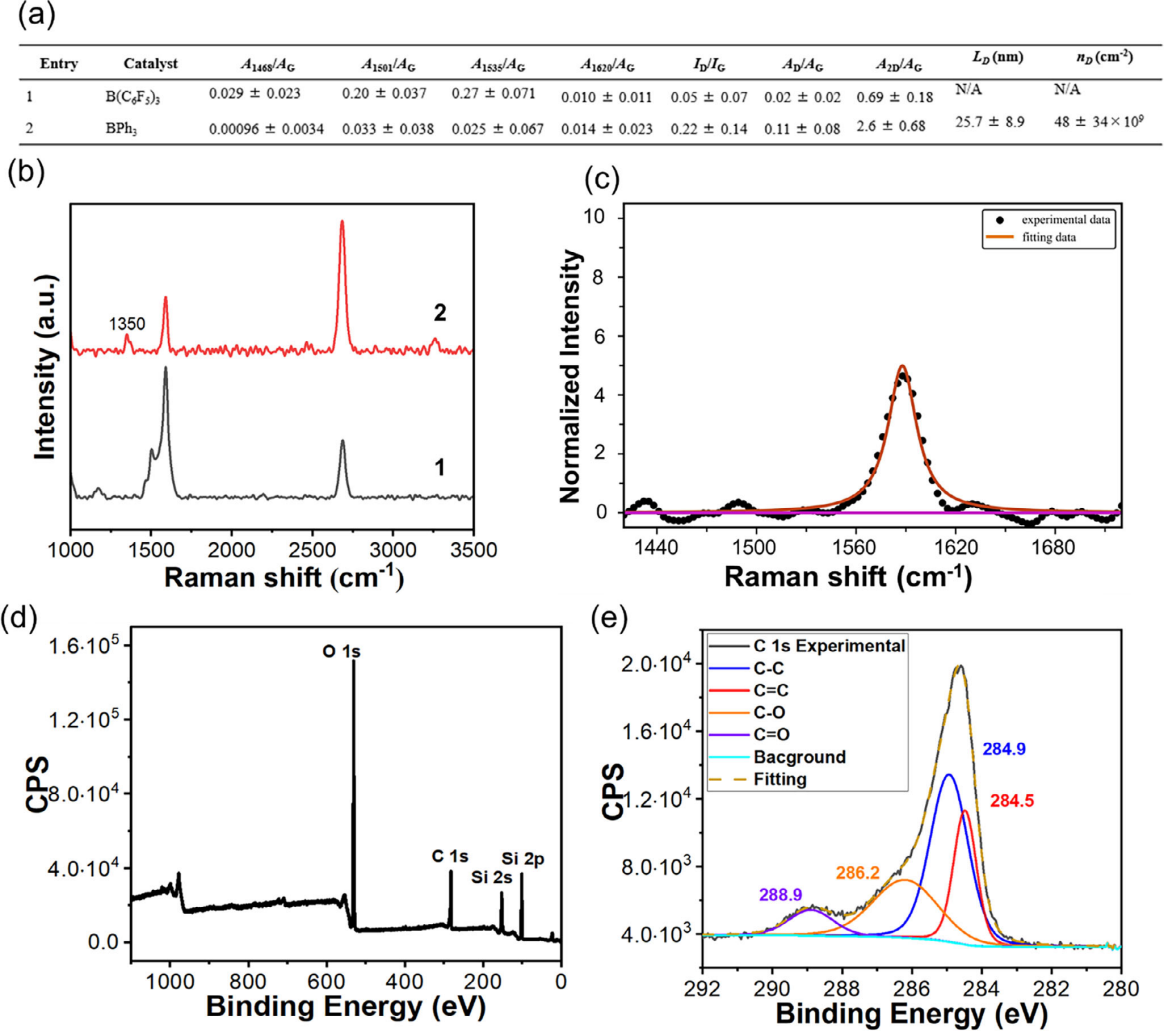
Reaction of tropone with Gra/Cu(111) using BPh_3_ as the catalyst. a) Raman data summary of reaction products and comparison with B(C_6_F_5_)_3_. Reactions were carried out at 50 °C for 1 h under neat condition without solvent. b) Typical Raman spectra of reaction products. All Raman spectra of entry 2 are presented in Figure S9a (Supporting Information), and data analysis in Figure S9b–d (Supporting Information). c) Raman G band deconvolution of entry 2. d) XPS survey scan and e) high‐resolution C 1s spectrum and peak deconvolution of entry 2.

To investigate the impact of Lewis acid on the cycloaddition pathway, BPh_3_ was used in the reaction of graphene with tropone (**Figure** **6**a, entry 2). The product was characterized and compared to the reaction using B(C_6_F_5_)_3_ as the catalyst under identical conditions (Figure 6a, entry 1). In contrast to the reaction catalyzed by B(C_6_F_5_)_3_, the G peak remained unchanged in graphene after reaction with tropone catalyzed by BPh_3_ (Figure 6b). Peak deconvolution yielded no additional peaks (Figure 6c), and quantitative analysis showed an order of magnitude lower *A*_1501_/*A*_G_ and *A*_1535_/*A*_G_ than B(C_6_F_5_)_3_ (Figure 6a, Figure S9b, Supporting Information). Additionally, the D peak at 1350 cm^−1^ appeared in reaction catalyzed by BPh_3_ (Figure 6b). The intensity ratios of D and G peak, *I*_D_/*I*_G_ or *A*_D_/*A*_G_, were 0.22 ± 0.14 and 0.11 ± 0.08, respectively (Figure 6a, Figure S9d, Supporting Information). These are significantly higher than tropone‐functionalized graphene using B(C_6_F_5_)_3_ as the catalyst under the same reaction conditions, at 0.05 ± 0.07 and 0.02 ± 0.02 for *I*_D_/*I*_G_ and *A*_D_/*A*_G_, respectively, which are similar to unfunctionalized pristine graphene (Figure 6a, entry 1). From the *I*_D_/*I*_G_ value, the defects were quantified by calculating the distance between two point defects, *L*_D_, and the defect density *n*_D_ (i.e., the number of point defects per cm^2^) using the method developed by Cançado et al.^[^ ^49^ ^]^ The results were 25.7 ± 8.9 nm and (48 ± 34) ×10^9^ cm^−2^ for *L*_D_ and *n*_D_, respectively (see SI for calculations).

The absence of changes in the G band and appearance of the D band indicate that the reaction catalyzed by BPh_3_ is significantly different than the one catalyzed by B(C_6_F_5_)_3_. To gain more insight into the reaction, XPS of BPh_3_‐catalyzed product was collected. The XPS survey scan contained the C 1s and O 1s peaks as expected (Figure 6d). In the high‐resolution C 1s spectrum, a new peak at 286.2 eV, which can be assigned to C─O,^[^ ^13^, ^56^, ^57^ ^]^ appeared (Figure 6e). This peak was absent in the product catalyzed by B(C_6_F_5_)_3_ (Figure 4g). In the [8 + 2] cycloaddition of tropone with graphene, an ether group is produced (Scheme 1b), whereas the [4 + 2] cycloaddition introduces a carbonyl group (Scheme 1a). For the BPh_3_‐catalyzed product, the peak intensity of C─O was much higher than that of C═O, at a ratio of 21 to 8.0. Based on these results, we hypothesize that the reaction of graphene with tropone catalyzed by BPh_3_ is dominated by the [8 + 2] pathway, whereas the reaction catalyzed by B(C_6_F_5_)_3_ prefers the [4 + 2] pathway.

**Figure 7:**
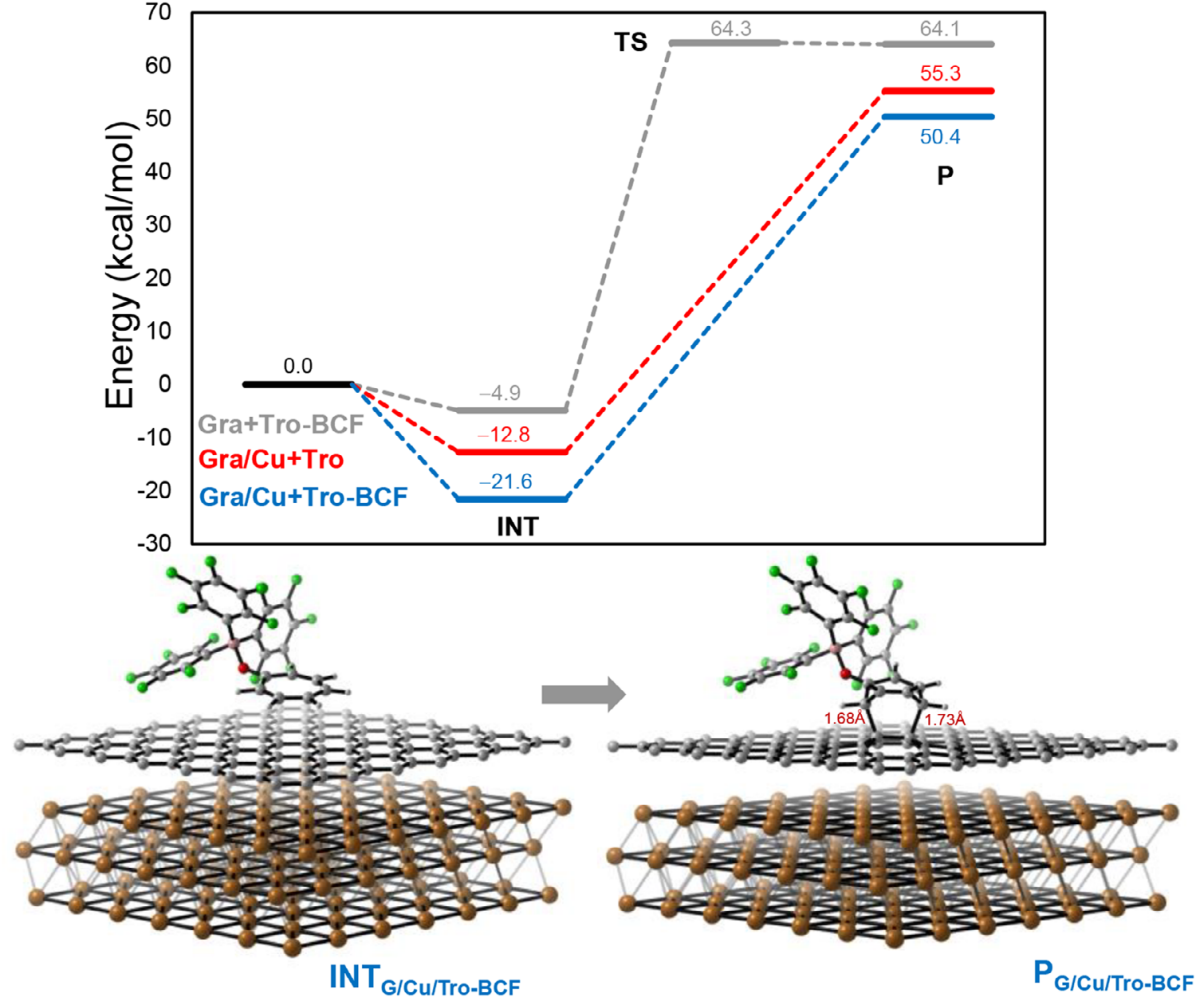
Reaction energy profiles of the [4 + 2] reaction involving graphene with tropone‐BCF (gray), graphene/Cu(111) with tropone (red), and graphene/Cu(111) with tropone‐BCF (blue).

To understand the reaction and selectivity in further detail, periodic DFT calculations were performed initially on a flat 6 × 6 graphene sheet with and without a Cu(111) substrate and Lewis acid (**Figure** **7**). The influence of the B(C_6_F_5_)_3_ was also pursued. The initial state includes the graphene sheet with or without Cu(111) and the tropone‐BCF complex at infinite separation which are then allowed to interact to form **INT**. Whether both Cu(111) and B(C_6_F_5_)_3_ are included or not, the thermodynamics for product formation are quite unfavorable with 50.4 kcal mol^−1^ being the least unfavorable in the **Gra/Cu/Tro‐BCF** case. A transition state for the **Gra/Tro‐BCF** path was located but was only 0.2 kcal mol^−1^ higher in energy than the product, highlighting the high kinetic and thermodynamic instability in the predicted product. The [8 + 2] product could not be located as a stable product with or without the inclusion of Cu(111) or a Lewis acid. While this analysis does suggest the combination of the Cu(111) substrate with the Lewis acid may lower energetics than in their absence, the energetics are unfavorable, and an approach that might lead to more favorable energetics was pursued.

**Figure 8:**
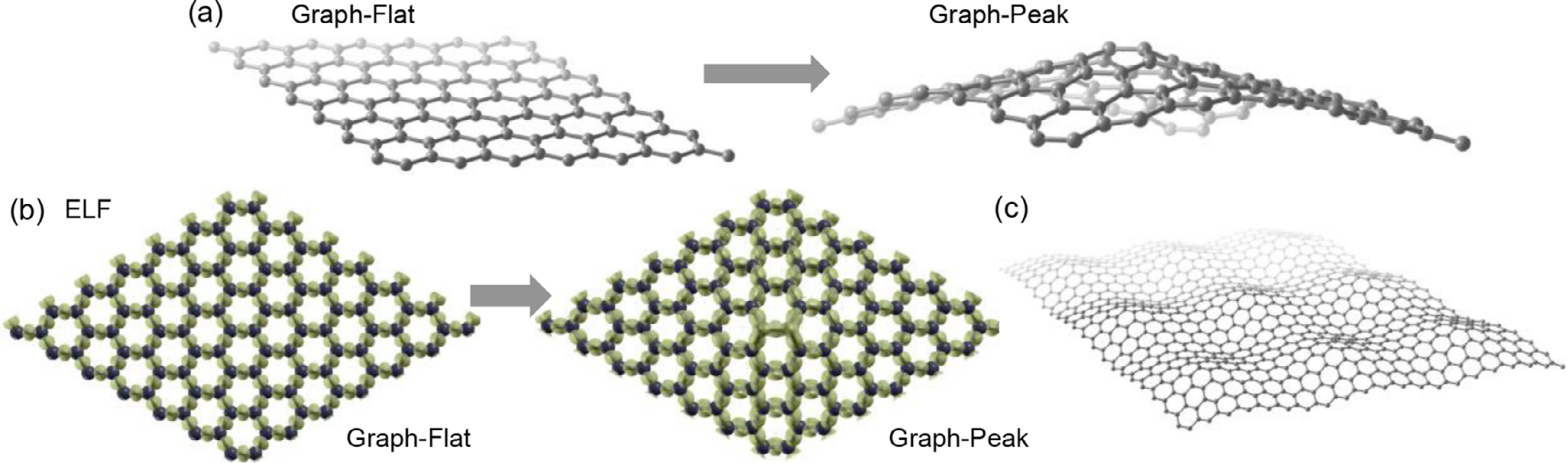
a) Comparison of flat graphene to peak graphene used for mechanism determination. b) Electron localization function (ELF) for graphene‐flat and graphene‐peak (ELF = 0.85). c) 3 × 3 peak graphene supercell provided for periodicity perspective.

Since the reaction exhibited quite unfavorable reaction energetics on a flat graphene sheet supported by Cu(111), a sheet reflecting potential imperfections and roughness of a substrate was sought that could lead to more favorable reaction energetics. Substrate roughness may impart peak‐like curvature on graphene, potentially leading to increased reactivity. Computationally, the peak curvature was maintained through limited atomic constraints along the z‐axis involving 8 graphene carbons which included the central two carbon atoms and the atoms at the corners of the supercell (Figure S12, Supporting Information). A separation distance along the z‐axis of 3.3Å was fixed between the central carbons and the peripheral carbons, causing pyramidalization in the two central carbons. This was achieved by successively increasing the z‐coordinate of the central two carbons while performing a series of variable‐cell geometry optimizations. This procedure produced a graphene sheet with peak‐like structure (**Figure** **8**a,c) with pyramidalization angle at the central carbons of 113.2°. The electron localization function (ELF) highlights significant localization of e‐density at the central carbons, suggesting enhanced reactivity (Figure 8b). A similar strategy has been applied to model curvature‐induced ring migration on curved graphene.^[^ ^72^ ^]^

**Figure 9:**
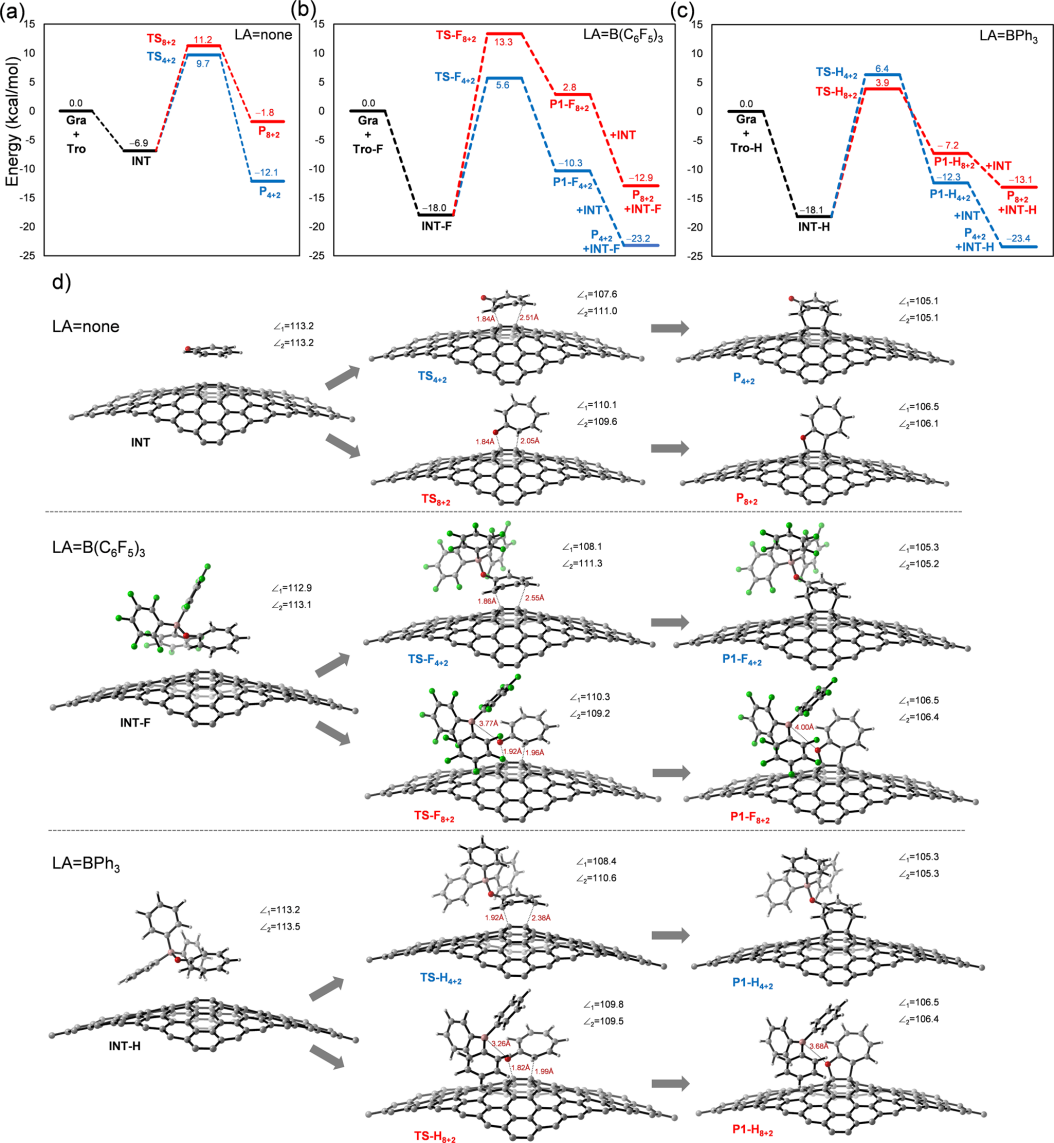
Reaction energy profiles for the [4 + 2] and [8 + 2] reactions a) without Lewis acid, b) with B(C_6_F_5_)_3_, c) with BPh_3_. d) Geometries of INT, TS, and P1 for each mode. Angles of pyramidalization are provided, where ∠_1_ is the angle of the left peak graphene C and ∠_2_ is the angle of the right peak graphene C.

Peak graphene was then used for the modeling of tropone reactivity excluding effects from a substrate (**Figure** **9**). In the absence of a Lewis acid, tropone first associates with graphene to form the physisorbed complex, **INT** (Figure 9a). **INT** is then transformed to the product through either a [4 + 2] or [8 + 2] reaction, **P**_4 + 2_, **P_8 + 2_**, with reaction barriers of 17.6 and 18.1 kcal mol^−1^ respectively. While the [4 + 2] reaction has only a slightly lower barrier than that of the [8 + 2] reaction, **P**_4 + 2_ is more stable by 10.3 kcal mol^−1^. While **INT** is predicted to be the lowest energy point toward product, the inclusion of entropic and solvation effects would lower the energy of the initial state below **INT**, thus increasing the activation barrier.

Including the B(C_6_F_5_)_3_ Lewis acid results in a much more stabilized complexation in **INT‐F** to −18.0 kcal mol^−1^, likely from both van der Waals and charge transfer interactions (Figure 9b). As in the uncatalyzed case, the inclusion of solvation and entropic effects would likely lower the total energy of the initial state to at or below that of **INT‐F**. From **INT‐F**, the [4 + 2] barrier is significantly lower than the [8 + 2] barrier (7.7 kcal mol^−1^), which is 6.2 kcal mol^−1^ more than in the uncatalyzed case. This leads to the initial products **P1‐F** with the Lewis acid still present, which is higher in energy than **INT‐F**. The next likely step would involve the transfer of the Lewis acid to another tropone (**INT)** leading to the uncomplexed product, resulting in a net thermodynamically favorable reaction for the [4 + 2] but slightly uphill in energy for the [8 + 2], relative to **INT‐F**.

Switching the Lewis acid to BPh_3_ (Figure 9c) leads to a similar stabilization in **INT‐H**. However, the transition states now shift in preference with the [8 + 2] having a lower activation energy by 2.5 kcal mol^−1^, leading to the initial products **P1‐H**, followed by **P_8 + 2_**_._ While **P_8 + 2_** is still higher in energy than **INT‐H** by 5.0 kcal mol^−1^, this can be reduced by starting from even greater pyramidalization in the starting graphene peak carbons through increased starting relative heights, which can be caused by greater roughness in the substrate. Any substrate‐induced charge transfer effects to graphene will also likely contribute to more favorable energetics, as suggested from product stabilization (Figure 7), that are not included here. A strongly donating substrate, like Ni(111), would be expected to lead to more favorable reactivity.

The TS energy difference in the BPh_3_ case (2.5 kcal mol^−1^) is significantly lower than the TS energy in the B(C_6_F_5_)_3_ case (7.7 kcal mol^−1^), suggesting that some minor [4 + 2] product might be observed in the BPh_3_ case while only [4 + 2] product would be observed in the B(C_6_F_5_)_3_ case. This conclusion appears to be consistent with the XPS data showing some minor C═O signal (288.9 eV), indicating minor [4 + 2] product present, in the reaction with BPh_3_ and no C─O signal (286.2 eV), indicating [8 + 2] product, in the reaction with B(C_6_F_5_)_3_.

The origin of the selectivity inversion can be better understood upon inspection of the respective reaction paths. For the [4 + 2] path, the Lewis acid is bound to the oxygen in the product using both Lewis acids as the oxygen is not involved in bond formation. For traversing the [8 + 2] path, the Lewis acid must dissociate prior to C─O bond formation (Figure 9b). In **TS‐F_8 + 2_**, the O‐B bond elongates to 3.77Å, with very little pyramidalization on the boron. Since the energy of O‐B dissociation is included on the reaction path, the path with the weaker O‐B bond energy will contribute less to the activation energy, which is BPh_3_ as it is the weaker Lewis acid. To assess whether the dissociation requirement is related to steric or electronic effects, the [8 + 2] product with BF_3_, a much smaller Lewis acid, was subjected to a geometry optimization. The resulting optimization led to a product with a similarly large O‐B distance as compared with **P1‐H_8 + 2_,** indicating the dissociation requirement is likely electronic in nature, related to low Lewis basicity on the ether product oxygen thereby reducing its coordination strength. The role of BPh_3_ in the [8 + 2] reaction may be a combination of providing electrostatic stabilization of an intermediate charge‐separated state on the reaction path and advancing partial C─C bond formation pre‐transition state, before O‐B dissociation.

While the initial graphene constraints produce pyramidalization suitable for a favorable reaction, the extent of pyramidalization is closer to flat graphene than to the product, indicating substantial pyramidalization during the reaction is still required. Furthermore, the z‐direction constraints do not hinder natural asynchronization in the reactions. The [4 + 2] reaction is more asynchronous as revealed in both bond lengths and bond angles of the TS's. Previous computational studies on Lewis acid‐catalyzed tropone cycloadditions revealed stepwise mechanisms,^[^ ^69^, ^70^ ^]^ in contrast to the concerted asynchronous mechanisms located here. The stepwise mechanisms observed in these studies is likely a consequence of using a highly polarized dienophile, 1,1‐dimethoxyethylene, that can lead to stabilization of charge‐separated intermediates, whereas such charge stabilization would be substantially reduced with graphene.

## 4 Reduction of Tropone‐Functionalized Graphene

**Figure 10:**
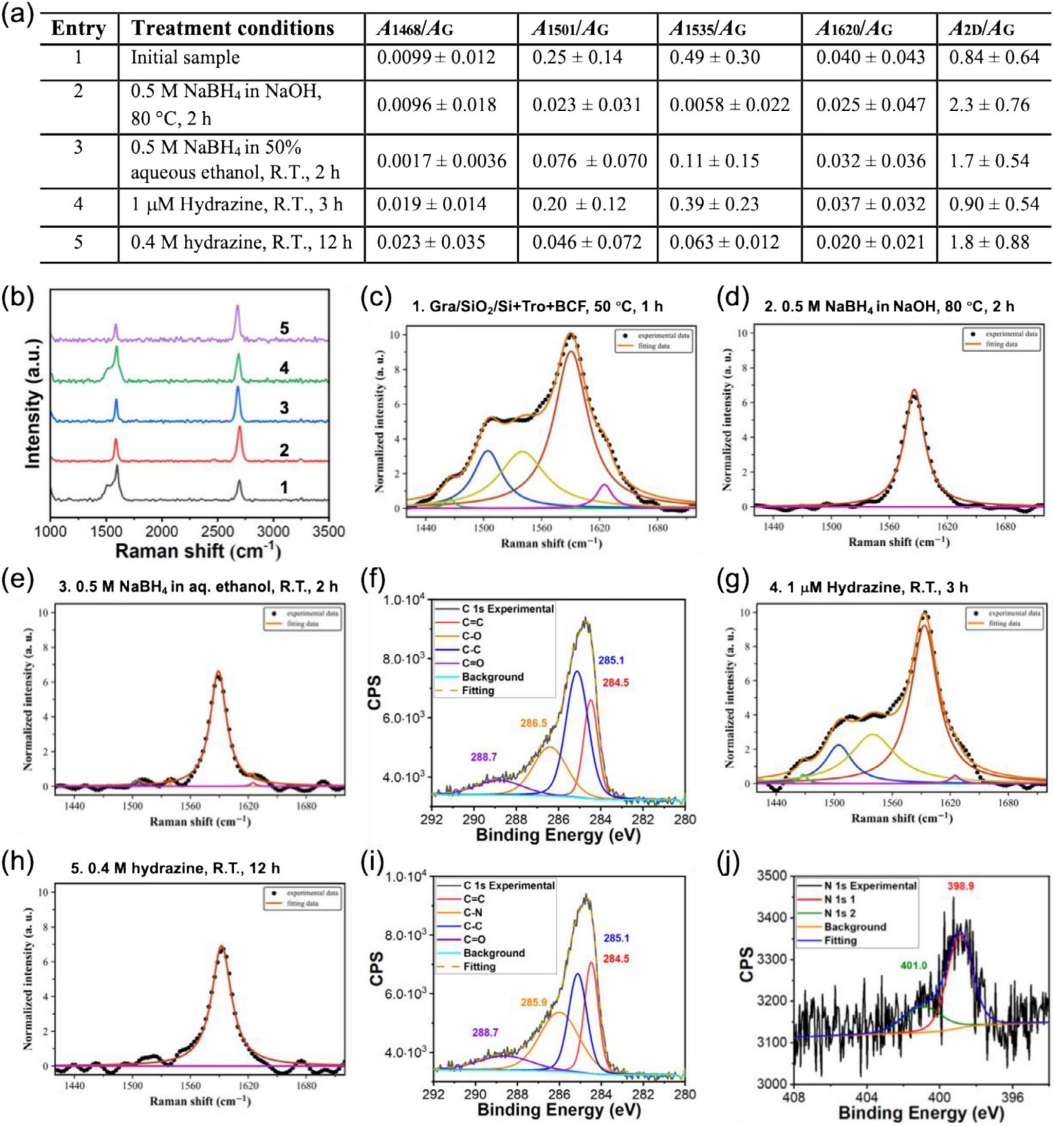
Reduction of tropone‐functionalized graphene with NaBH_4_ or hydrazine. a) Samples, reagents and conditions, and Raman data summary. The initial sample was prepared by treating Gra/SiO_2_/Si with tropone and B(C_6_F_5_)_3_ at 50 °C for 1 h. b) Representative Raman spectra of samples in (a). All Raman spectra can be found in Figure S10 (Supporting Information). d,e) Raman G band deconvolution of tropone‐functionalized graphene after reduction with NaBH_4_ (entries 1, 2). f) High‐resolution XPS spectrum and deconvolution of C 1s peak of sample after reduction with NaBH_4_ (entry 3). g,h) Raman G band deconvolution of sample after reduction with hydrazine (entries 4, 5). High‐resolution XPS spectrum and deconvolution of i) C 1s peak and j) N 1s peak of sample after reduction with hydrazine (entry 5).

The cycloaddition reaction catalyzed by B(C_6_F_5_)_3_ introduces carbonyl groups on graphene. To further test the presence of carbonyl groups, tropone‐functionalized graphene was treated with a reducing agent, NaBH_4_^[^ ^73^, ^74^, ^75^, ^76^ ^]^ or hydrazine.^[^ ^77^, ^78^, ^79^ ^]^ Tropone‐functionalized graphene was prepared by reacting tropone with graphene supported on silicon wafer in the presence of B(C_6_F_5_)_3_ at 50 °C for 1 h (a, entry 1). For NaBH_4_, two different conditions were used: in pH 9.5 NaOH (entry 2), which is commonly used for reducing GO,^[^ ^75^ ^]^ and in 50% aqueous ethanol (entry 3), which is commonly used for reducing carbonyl groups in organic compounds.^[^ ^74^ ^]^ The shoulder peaks on the Raman G band, which were present in the initial sample (**Figure** **10**c), disappeared after treating with NaBH_4_/NaOH (Figure 10d) or NaBH_4_/ethanol (Figure 10e). *A*_2D_/*A*_G_ increased significantly, more so in NaOH to 2.3 ± 0.76 than in ethanol to 1.7 ± 0.54 (Figure 10a, entries 2 & 3). The noticeable decrease in G band shoulder peaks and increase in *A*_2D_/*A*_G_ after reduction are similar to those observed when GO was reduced to rGO.^[^ ^46^, ^75^, ^80^ ^]^ Treating unfunctionalized graphene with NaBH_4_/NaOH (Figure S10h, Supporting Information) or NaBH_4_/ethanol (Figure S10i, Supporting Information) did not change the Raman characteristics of the G band. The reduction reaction product was further characterized by XPS. After treating with NaBH_4_, a peak at 286.5 eV, which was absent in the tropone‐functionalized graphene (Figure 4g), appeared (Figure 10f). This peak can be assigned to the C─O group.^[^ ^81^ ^]^ Furthermore, the intensity of the C═O peak decreased from 14% in tropone‐functionalized graphene to 5.6% after treating with NaBH_4_ (Table S4, Supporting Information). These results are consistent with the reduction of the carbonyl groups in tropone‐functionalized graphene by NaBH_4_.

For the reduction with hydrazine, tropone‐functionalized graphene was first treated with 0.13 µm hydrazine, which amounted to 1.2 equivalents of carbonyl assuming that tropone fully reacted with graphene to form a monolayer product. After 3 h at room temperature, the shoulder peaks decreased slightly (entry 4 in Figure 10a,g). The sample was then treated with 0.4 M hydrazine at room temperature for 12 h. In this case, the shoulder peaks disappeared completely (Figure 10h), and *A*_2D_/*A*_G_ increased to 1.8 ± 0.88 (Figure 10a, entry 5). Treating the unfunctionalized graphene with 0.4 M hydrazine at room temperature for 12 h did not change the Raman characteristics of the G band (Figure S10j, Supporting Information). Following hydrazine treatment, two peaks at 398.9 and 401.0 eV, respectively, appeared in the high‐resolution XPS N 1s spectrum (Figure 10j). The peak at 398.9 eV was observed in both tropone‐functionalized graphene (Figure S11a, Supporting Information) and after treating with NaBH_4_ (Figure S11b, Supporting Information). This peak may originate from residual N_2_ gas from the atmosphere that is physisorbed on graphene surface, particularly when samples are stored under ambient conditions.^[^ ^82^ ^]^ Importantly, the N 1s peak at 401.0 eV is absent in neither tropone‐functionalized graphene (Figure S11a, Supporting Information) nor after treating with NaBH_4_ (Figure S11b, Supporting Information). Although the exact nature of the *N*‐containing functional group is difficult to confirm, it can be undoubtedly assigned to nitrogen species. Additionally, a signal at 285.9 eV appeared in the C 1s spectrum after treating with hydrazine (Figure 10i). Considering the concurrent presence of the N species, this peak can be attributed to C─N/C═N structure in graphene.^[^ ^81^ ^]^

## 5 Conclusion

We have successfully demonstrated the functionalization of graphene using tropone as an electron‐deficient diene and graphene supported on Cu(111) as an electron‐rich dienophile via the IEDDA mechanism under mild conditions. The reaction was enhanced by a Lewis acid, which increases the electron deficiency of tropone by lowering its LUMO. The synergistic activations of graphene through Cu(111)‐mediated electron donation and of tropone via Lewis acid coordination collectively enhance the HOMO–LUMO interactions to drive the IEDDA cycloaddition reactions.

The reaction using B(C_6_F_5_)_3_ as the catalyst resulted in the appearance of shoulder peaks of the graphene Raman G band, and upon deconvolution, yielded the D″ bands at 1501 and 1535 cm^−1^ as the most significant peaks. Increasing the electron deficiency using chlorotropone resulted in increased shoulder peaks, hinting higher extent of graphene functionalization. The DFT calculations support favorable reaction energetics through Lewis acid activation on curved graphene. XPS confirmed the presence of the carbonyl group in the product and additional Cl peak in the case of chlorotropone, supporting the successful functionalization of graphene. Similar results were obtained for graphene supported on silicon wafer, owing to substrate‐induced electron puddles which are known to enhance the reactivity of graphene. Treating tropone‐functionalized graphene with a reducing agent NaBH_4_ or hydrazine resulted in the complete disappearance of the shoulder peaks in the Raman G band, reduced C─O peak in the XPS spectra, and appearance of nitrogen species in the XPS of hydrazine‐treated sample.

Surprisingly, using BPh_3_ instead of B(C_6_F_5_)_3_ as the Lewis acid catalyst gave no shoulder peaks in the G band, but rather the appearance of the D band. XPS showed the appearance of a significant amount of C─O, which is absent in the B(C_6_F_5_)_3_‐catalyzed product. This result supports the preference of a [8 + 2] cycloaddition mechanism with BPh_3_ which gives an ether product, in contrast to the [4 + 2] cycloaddition mechanism with B(C_6_F_5_)_3_ which gives a ketone product. The DFT predicted mechanism reveals the origin of this Lewis acid‐dependent selectivity inversion to be based on required Lewis acid dissociation, with the stronger Lewis acid, B(C_6_F_5_)_3_, requiring more activation energy for dissociation than with BPh_3_. Using substrate to enhance the reactivity of graphene and a specific catalyst to modulate the reaction pathways represents a new strategy for expanding the currently limited scope of graphene chemistry.

## 6 Experimental Section

### Instrumentation

Raman measurements were performed at room temperature on a Raman spectrometer (Bruker Senterra I) at 532 nm and 2 mW incident power. The irradiation time was 10 s. Previously, systematic studies were conducted to show that the laser irradiation under these conditions does not cause sample damage or introduce additional peaks in the Raman spectra.^[^ ^34^, ^83^ ^]^ Spectra were collected at random locations across the sample. Previous studies demonstrated that averaging multiple random spots yielded results consistent with Raman imaging.^[^ ^34^ ^]^^1^H NMR spectra were collected on a JEOL ECZ 400 MHz spectrometer. IR spectra were collected on a FI‐IR spectrometer (PerkinElmer, Spectrum Two). XPS was conducted on PHI Genesis, and data were analyzed with CasaXPS.

### Solvent‐Free Reaction of Tropone with Graphene on Cu(111) or Silicon Wafer

To a piece of ≈0.8 × 2 cm CVD graphene on Cu(111) on a hot plate, a drop of B(C_6_F_5_)_3_ (26.4 mg, 0.0515 mmol, 5.00 mol% based on tropone, TCI, >98%) dissolved in tropone (100 µL, 1.03 mmol, 1 eq, Thermo Scientific, 97%) was added to cover the entire graphene surface. The sample was then covered with a petri dish and was either left at room temperature or heated at 50 °C for 1, 2, or 10 h. Afterward, the sample was soaked in toluene (Fisher Chemical, certified ACS) two times, THF (Fisher Chemical, 99.9% in assay) two times, and acetone (Fisher Chemical, ACS grade) three times. After drying, it was transferred onto a silicon wafer (280 nm thick SiO_2_ layer, Fuleda Technology) for Raman characterization. Graphene on silicon wafer was prepared by transferring Gra/Cu(111) onto a silicon wafer following the previous procedure,^[^ ^84^ ^]^ see Supporting Information for details.

Reactions without B(C_6_F_5_)_3_ or tropone, or solvent‐free reaction using BPh_3_ were carried out following the same procedure described above except the following:

### Without B(C_6_F_5_)_3_

Tropone (≈20 µL) was deposited on Gra/Cu(111) and was heated on a hot plate at 50 °C for 2 h.

### Without Tropone

A drop of B(C_6_F_5_)_3_ (0.240 g, 0.470 mmol) dissolved in 1 mL toluene was deposited on Gra/Cu(111), and was heated at 50 °C on a hot plate for 31 h.

### Solvent‐Free Reaction Using BPh_3_

BPh_3_ (24.2 mg, 0.100 mmol, 10.0 mol% based on tropone, Thermo Scientific, 96%) dissolved in tropone (100 µL, 1.03 mmol, 1 eq) was deposited on Gra/Cu(111) and was heated on a hot plate at 50 °C for 1 h.

### Reaction of Tropone or 2‐Chlorotropone with Graphene in Toluene

To tropone (31.8 mg, 0.300 mmol, 1 eq) or 2‐chlorotropone (42.2 mg, 0.300 mmol, 1 eq) in 1 mL toluene was added B(C_6_F_5_)_3_ (15.4 mg, 0.0300 mmol, 10 mol%). A piece of ≈0.8 cm × 2 cm Gra/Cu(111) was placed in the solution. The reaction was carried out at room temperature for 3 h under shaking or heated at 50 °C for 31 h. Afterward, the sample was soaked in toluene two times, THF two times, and acetone three times. After drying, functionalized graphene was transferred onto a silicon wafer for Raman characterization.

### Reduction of Tropone‐Functionalized Graphene

The initial samples were prepared by reacting graphene on silicon wafer, ≈0.5 cm × 0.5 cm in size, with tropone in the presence of B(C_6_F_5_)_3_ at 50 °C for 1 h. Controls were unfunctionalized graphene on silicon wafer treated in the corresponding reduction solutions following the same procedure. All samples were dried with N_2_ gas before characterization.

### Reduction with NaBH_4_/NaOH

A solution of 500 mm NaBH_4_ in NaOH was prepared by dissolving NaBH_4_ (189 mg, Fluka ≥99%) in an aqueous NaOH solution (10 mL, ≈0.1 mm, pH ≈9.5). Graphene sample was placed in 10 mL of this solution in a beaker and was heated at 80 °C for 2 h. Afterward, the sample was washed with Milli‐Q water (resistivity 18.2 MΩ·cm) three times, toluene two times, THF two times, and acetone three times.

### Reduction with NaBH_4_/Ethanol

A solution 500 mm of NaBH_4_ in ethanol was prepared by dissolving NaBH_4_ (189 mg) in 10 mL 50% aqueous ethanol, prepared by mixing equal volume of 200 proof ethanol with Milli‐Q water. Graphene sample was placed in the solution at room temperature for 2 h. Afterward, the sample was washed with Milli‐Q water three times, toluene two times, THF two times, and acetone three times.

### Reduction with Hydrazine

A 0.13 µm aqueous solution of hydrazine was prepared by diluting N_2_H_4_·H_2_O (TCI, >98.0%) with Milli‐Q water. Graphene sample was placed in this solution (5 mL, ≈1.2 eq of calculated maximal tropone‐functionalized graphene assuming graphene area of 0.5 cm × 0.5 cm) in a beaker and was shaken on a shaker (60 rpm min^−1^) at room temperature for 3 h. The sample was washed with Milli‐Q water three times and dried. After Raman characterization, 100 µL N_2_H_4_·H_2_O was added to the above solution, and the same sample was shaken in the solution at room temperature for 12 h. The sample was washed with Milli‐Q water three times and dried.

### Computational Method

All DFT calculations were performed using the Plane‐Wave Self‐Consistent Field (PWSCF) plane wave code using the Quantum Espresso software package.^[^ ^85^ ^]^ The generalized gradient approximation (GGA) was applied using the Perdew–Burke–Ernzerhof (PBE)^[^ ^86^ ^]^ functional augmented with the DFT‐D3 dispersion correction.^[^ ^87^ ^]^ Ultrasoft pseudopotentials were used. A Gaussian smearing parameter of 0.002 Ry was used for Brillouin‐zone integration in calculations using Cu. All geometry optimizations were performed using a Monkhorst‐pack 1 × 1 × 1 *k‐*point grid while all final reported SCF energies and electron densities were determined using a 3 × 3 × 1 *k*‐point grid. Kinetic energy cutoffs for the charge density and wavefunctions of 400 Ry and 40 Ry respectively were used. The supercell consisted of a 6 × 6 graphene sheet with and without a three‐layer Cu slab with the bottom Cu slab layer fixed in the z‐direction for all geometry optimizations. The peak structure of graphene was maintained by fixing the central 2 carbons and peripheral 8 carbons at the box corners in the z‐direction while performing variable‐cell optimizations by successively increasing the z‐coordinate for the central two carbons until a separation of 3.3 Å between the central 2 carbons and peripheral carbons was reached in the z‐direction. A vacuum spacing of ≈15Å was applied. All transition states and minimum energy paths were located using the climbing image elastic band (CI‐NEB) method.^[^ ^88^, ^89^ ^]^

## Conflict of Interest

The authors declare no conflict of interest.

## Supporting information



Supporting Information

## Data Availability

The data that support the findings of this study are available from the corresponding author upon reasonable request.
