# Supplementary material for: Inverse‐Electron‐Demand Diels–Alder Reaction of Tropone with Graphene Supported on Cu(111)
Source: Small. 2025 Sep 30;21(44):e03669. doi: 10.1002/smll.202503669 (PMC12590532; doi:10.1002/smll.202503669)
Supplement: Supplementary file 1 — Supporting Information [file SMLL-21-e03669-s001.pdf]

NANO · MICRO small

Supporting Information

for *Small*, DOI 10.1002/smll.202503669

Inverse-Electron-Demand Diels–Alder Reaction of Tropone with Graphene Supported on Cu(111)

Jia Tu, Wentong Zhou, Lawrence M. Wolf and Mingdi Yan**

Supporting Information

Inverse-Electron-Demand Diels-Alder Reaction of Tropone with Graphene Supported on Cu(111)

Jia Tu, Wentong Zhou, Lawrence M. Wolf Mingdi Yan**
University of Massachusetts Lowell, Lowell, MA 01854

Table of Contents

1. Literature Summary of Diels-Alder (DA) Reactions on Graphene S1
2. Transfer graphene on Cu(111) to silicon wafer S2
3. Additional Raman spectra of Gra/Cu(111) after reaction with tropone and control samples S3
4. Reaction of Tropone with Ethyl Vinyl Ether and Product Characterization S4
5. Additional Raman Spectra of Compound 1 and Tropone+5%$B(C_6F_5)_3$ on Graphene S5
6. Testing Cycloreversion of Tropone-Functionalized Graphene S6
7. Effect of Temperature and Time on the Reaction of Gra/Cu(111) with Tropone Catalyzed by $B(C_6F_5)_3$: All Raman Spectra and Data Analysis of Samples in Figure 3 .... S7
8. Zoomed-in XPS Spectra of Tropone-Functionalized Graphene in the Regions of B and F. S7
9. All Raman Spectra and Data Analysis of Entries 2–3 in Figure 5 .... S8
10. Reaction of Tropone with Gra/Cu(111) Catalyzed by BPh_3: All Raman Spectra and Data Analysis of Entry 2 in Figure 6 .... S9
11. Calculation of L_D and n_D S10
12. Treating Tropone-Functionalized Graphene with $NaBH_4$ or Hydrazine: All Raman Spectra, N1s XPS Spectra, and Data Analysis S11
13. DFT Energy Table S13
14. Peak Graphene Constraints S14
15. References S14

1. Literature Summary of Diels-Alder (DA) Reactions on Graphene

Table S1. Summary of DA reactions on graphene.

Reagent	Type of graphene	Reaction conditions	Extent of functionalization	Cycloreversion and conditions	Ref.
Tetracyanoethylene	Exfoliated graphene flakes (XG_{flake}) on silicon wafer<br>Exfoliated graphene (XG_{sol})<br>Highly oriented pyrolytic graphite (HOPG)	XG_{flake}: In 1,4-dioxane or dichloromethane, room temperature, 3 h<br>XG_{sol}: 0.05 M in 1,4-dioxane or dichloromethane, 45 °C, 3 h<br>HOPG: 0.05 M in 1,4-dioxane and dichloromethane, refluxing, 45 °C	XG_{flake}: D peak is most prominent in single-layer graphene<br>XG_{sol}: $I_D/I_G = 0.77$ (initial 0.03)<br>HOPG: $I_D/I_G = 0.22$ (initial: 0.00, edges: 0.05)	When the reaction was carried out at 100 °C in *p*-xylene, no functionalization was observed, indicating that the reaction is reversible at this temperature.	
Maleic anhydride	HOPG<br>Epitaxial graphene grown on SiC<br>XG_{sol}	HOPG: 0.15 M in *p*-xylene, 120 °C, 3 h<br>Epitaxial graphene: 0.25 M in *p*-xylene, 70 °C<br>XG_{sol}: 0.25 M in *p*-xylene, 130 °C, 3 h	HOPG: $I_D/I_G = 0.63$<br>Epitaxial graphene: I_D/I_G = 0.3 (initial 0.0)<br>XG_{sol}: $I_D/I_G = 0.65$ (initial 0.02)	Complete cycloreversion at 150 °C	[1]
9-Methylanthracene	HOPG<br>XG_{sol}	HOPG: 0.07 M in *p*-xylene, 130 °C<br>XG_{sol}: 0.1 M in *p*-xylene, 130 °C, 12 h	HOPG: I_D/I_G = 0.86-1.88<br>XG_{sol}: I_D/I_G = 1.37-1.63 (initial 0.02)	In *p*-xylene, 160 °C	
2,3-Dimethoxy-1,3-butadiene (DMBD)	Epitaxial graphene<br>XG_{sol}<br>HOPG	Epitaxial graphene: neat DMBD in argon, 50 °C, 3 h<br>XG_{sol}: 0.1 M in *p*-xylene, 120 °C<br>HOPG: 0.1 M in *p*-xylene, 120 °C	Epitaxial graphene: I_D/I_G = 0.5 (initial 0.0)<br>XG_{sol}: $I_D/I_G = 1.1$ (initial 0.0)<br>HOPG: $I_D/I_G = 0.38$ (initial 0.0)	DMBD-Epitaxial graphene: N/A<br>DMBD-XG_{sol}: 170 °C (I_D/I_G = 0.15)<br>DMBD-HOPG: 160 °C (I_D/I_G = 0.06)	
Raman-active cyanine 3, cyclopentadiene	Graphene on 285 nm SiO_2 silicon wafer	~100 mN force at each spot for 15-30 min	$I_D/I_G = 0.56$ (initial 0.16)	N/A	[2]
Iron phthalocyanine	Graphene epitaxially grown on Ir(111) substrate	Home-built scanning tunneling microscope (STM) at 5.2 K and base pressure < 10^{-8} Pa	Most reactive on top-fcc moiré registry of graphene	Electron removal from HOMO via STM tip at $V < -1.1$ V with sufficiently high currents *(I > 100* pA*)*	[3]
Cis-diene: dihydronaphthalene (D1)<br>Trans-diene: indene (D2)	CVD graphene transferred to silicon wafer	In reagent solution, room temperature D1: 5 min<br>D2: 15 min	D1: I_D/I_G = 0.4 – 0.6 for single crystal graphene.<br>D2: No discernible Raman spectral changes	D1: Good thermal stability even after annealed in Ar at 500 ˚C for 1 h<br>D2: N/A	[4]
Fluorinated maleimide	Epitaxial graphene on SiC(0001)	0.06 M in toluene at room temperature	I_D/I_G = 1.38 at 50 h, 1.60 at 80 h (initial: 0.3)	N/A	[5]
3,5-Bis(carboxylic acid)-phenyl-3-maleimide	Graphene epitaxially grown on Cu(111)	Irradiation (365 nm) in ultrahigh vacuum, room temperature, 8 min	I_D/I_G = 0.17 (initial: 0.068)	Reversible under extended UV irradiation, reaching equilibrium with ~56% yield at >30 min UV exposure	[6]
DMBD<br>Maleic anhydride (MAH)	Graphene transferred to Ni, Cu, or silicon wafer	Drop-cast reactant in *p*-xylene (0.1 M), heat at 50 °C, 100 °C, or 150 °C	DMBD: At 50 °C, Gra/Ni > Gra/Cu > Gra/SiO_2/Si<br>MAH: At 150 °C, Gra/Ni > Gra/Cu > Gra/SiO_2/Si	N/A	[7]

Table S2. Summary of computation results of DA reaction on graphene.

Reactants	Graphene Model	Electronic Binding Energy (Ref. 8) or Reaction Enthalpy (Refs. 2&9) (kcal mol^{-1})	Ref.
2,3-Dimethoxy-1,3-butadiene	4×4, 5×5, 6×6 Supercell	37.5, 47.3, and 46.3, respectively	[8]
9-Methyl-anthracene	5×5 Supercell	59.1	
9,10-Dimethyl-anthracene	5×5 Supercell	65.6	
Tetracyanoethylene	5×5 Supercell	63.2	
Maleic anhydride	5×5 Supercell	49.7	
Cyclopentadiene	5 × 5 Edge site (Bond a, b) and basal plane (Bond c)	-11.3, -1.4, and 36.6	[2]
2,3-Dimethoxy-1,3-butadiene	Peripheral bonds	-22.2 – -0.4	[9]
	Interior bonds	37.5 – 43.8	
9-Methylanthracene	Peripheral bonds	-9.2 – 4.9	
	Interior bonds	39.4 – 46.4	
Tetracyanoethylene	Peripheral bonds	-3.2 – 56.8	
	Interior bonds	42.5 – 46.8	
Maleic anhydride	Peripheral bonds	-9.8 – 37.0	
	Interior bonds	39.4 – 43.6	

2. Transfer graphene on Cu(111) to silicon wafer

This procedure applies to both as prepared CVD and functionalized graphene on Cu(111).
A solution of poly(methyl methacrylate) (PMMA, MW 996,000) in acetone (40 mg/mL) was spin-coated on Gra/Cu(111) at 1000 rpm for 1 min to form a protective film on graphene. Then, the sandwich of PMMA film, graphene, and the Cu(111) foil was added to the etching solution, 1 M $FeCl_3$ in 3 M HCl, for about 20 s. The sample was taken out and the graphene on the bottom of the Cu foil was wiped several times with a Kimwipe to remove the bottom graphene layer. The sample was put back into the etching solution for about 2 h, at which time the Cu foil was removed, and the sample became transparent. The graphene on PPMA was washed with 1 M HCl once and Milli-Q water three times. Then, a piece of silicon wafer (cleaned with ethanol and Milli-Q water) was immersed into the Milli-Q water to scoop the floating film onto the wafer. The sample was left in the air to dry for about 12 h until there was no observable water. Finally, the sample was soaked in acetone, twice for 3 h each, washed with acetone three times after each soaking to remove the PMMA layer.

3. Additional Raman spectra of Gra/Cu(111) after reaction with tropone and control samples

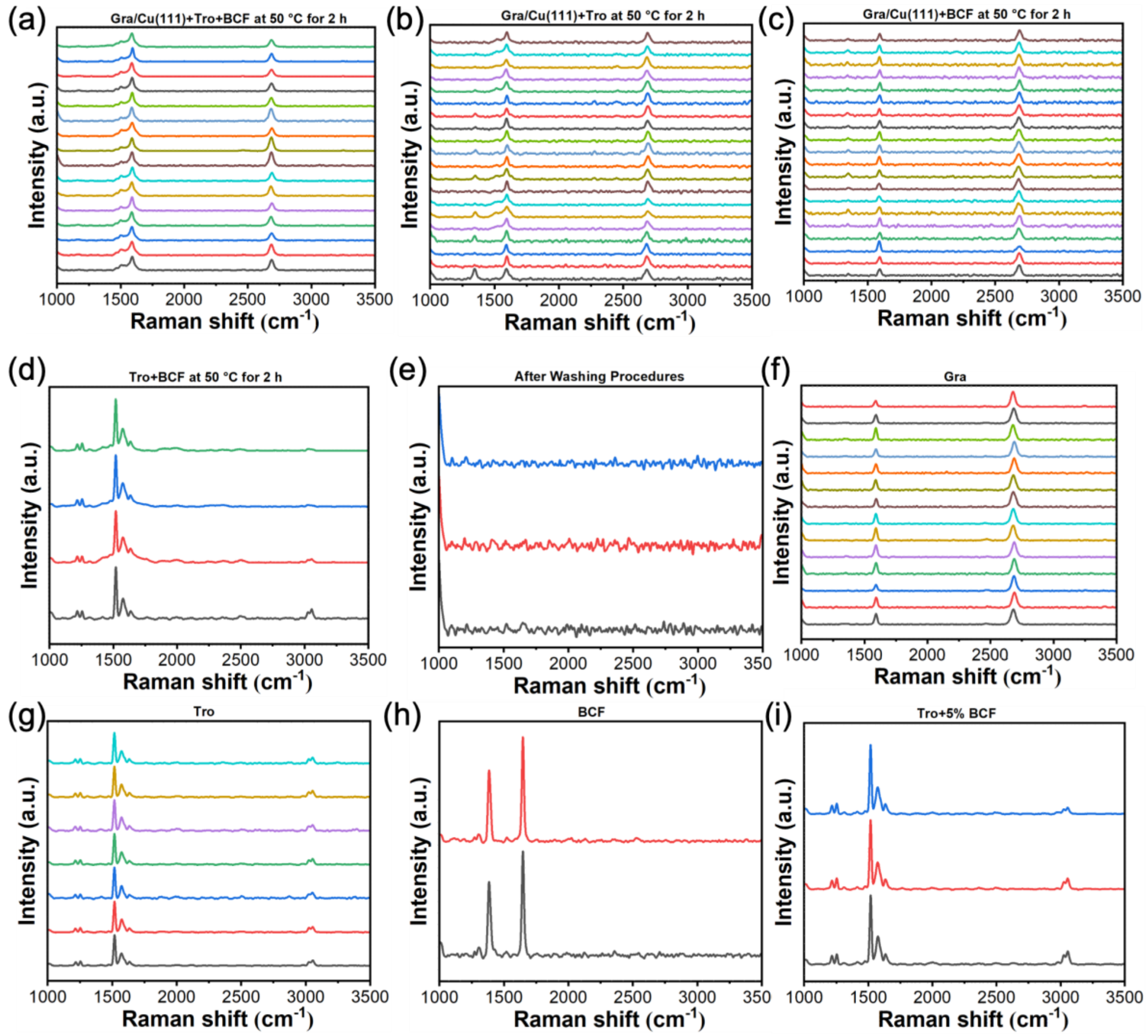

Figure S1. Raman spectra of (a) Gra/Cu(111) after reaction with tropone in the presence of $B(C_6F_5)_3$ at 50 °C for 2 h, (b) Gra/Cu(111) with tropone at 50 °C for 2 h, (c) Gra/Cu(111) with $B(C_6F_5)_3$ at 50 °C for 2 h, tropone and $B(C_6F_5)_3$ on silicon wafer after heating at 50 °C for 2 h (d) before and (e) after washing with solvents, (f) CVD graphene, (g) tropone, (h) $B(C_6F_5)_3$, and (i) tropone + 5% $B(C_6F_5)_3$. All spectra were taken on silicon wafers.

4. Reaction of Tropone with Ethyl Vinyl Ether and Product Characterization

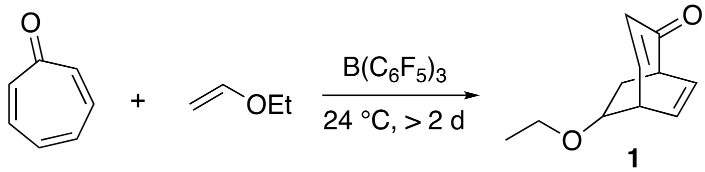

1

Scheme S1. Model reaction of tropone with ethyl vinyl ether to give compound **1**.

A literature procedure was followed.[10] To a solution of $B(C_6F_5)_3$ (46.2 mg, 0.0900 mmol) in tetrahydrofuran (THF, 3 mL) was added tropone (95.4 mg, 0.900 mmol) and ethyl vinyl ether (324 mg, 4.50 mmol). The reaction was stirred at 24 °C and was monitored by thin-layer chromatography (TLC) using 2.7:1 v/v hexanes:ethyl acetate. After 21 hours, starting materials were still present, particularly tropone. By 48 h, the amount of starting material decreased significantly and remained unchanged thereafter. The reaction was stopped at 120 h. Solvent was removed on a rotary evaporator at 35 °C to give the crude product as a dark brown liquid. Purification by column chromatography (3:1 v/v hexanes:ethyl acetate) gave **1** as a pale-yellow oil (50 mg, 31%). ^{1}H NMR (400 MHz, $CDCl_3$) δ 6.95 (dd, *J* = 11.1, 9.0 Hz, 1H), 6.44 (t, *J* =7.5 Hz, 1H), 6.24 (t, *J* = 8.0 Hz, 1H), 5.76 (dd, *J* = 11.1, 2.0 Hz, 1H), 4.01 (m, 1H), 3.59-3.42 (m, 4H), 2.36-2.27 (m, 1H), 1.91-1.82 (m, 1H), 1.18 (t, *J* = 7.0 Hz, 3H). IR (neat) ν_{max}/cm^{-1}: 3051, 2973, 2930, 2871, 1666, 1634, 1208, 1122, 1089, 1066, 1018, 914, 835, 713, 676.

The structure of **1** was confirmed by ^{1}H NMR (Figure S2a). In the IR spectrum, the peak at 1666 cm^{-1} corresponds to the C=O stretching within its conjugated system, and 1634 cm^{-1} corresponds to the isolated C=C bond. Both C=O and C=C peaks appear at lower wavenumbers in tropone at 1632 cm^{-1}, and 1567 and 1519 cm^{-1}, respectively, due to extended conjugation.[11] The C–O (ether) stretching observed at 1089 cm^{-1} appears only in **1** and not in tropone. The Raman spectrum of **1** contains characteristic peaks similar to those of its IR. The major IR and Raman bands and peak assignments of **1** and tropone are listed in Table S3.

Table S3. Major IR and Raman peaks of Compound **1** and tropone.

	**IR (cm^{-1})**		**Raman (cm^{-1})**	
	**Compound 1**	**Tropone**	**Compound 1**	**Tropone**
**C-H (*sp*^{2}) stretch**	3051	3022	3052	3053, 3024
**C-H (*sp*^{3}) stretch**	2973, 2930, 2871	Not observed	2876, 2934, 2966	Not observed
**C=O stretch**	1666	1632	1664	1634
**C=C stretch**	1634	1567, 1519	1622	1571, 1517
**C-O stretch**	1089	Not observed	1209	Not observed

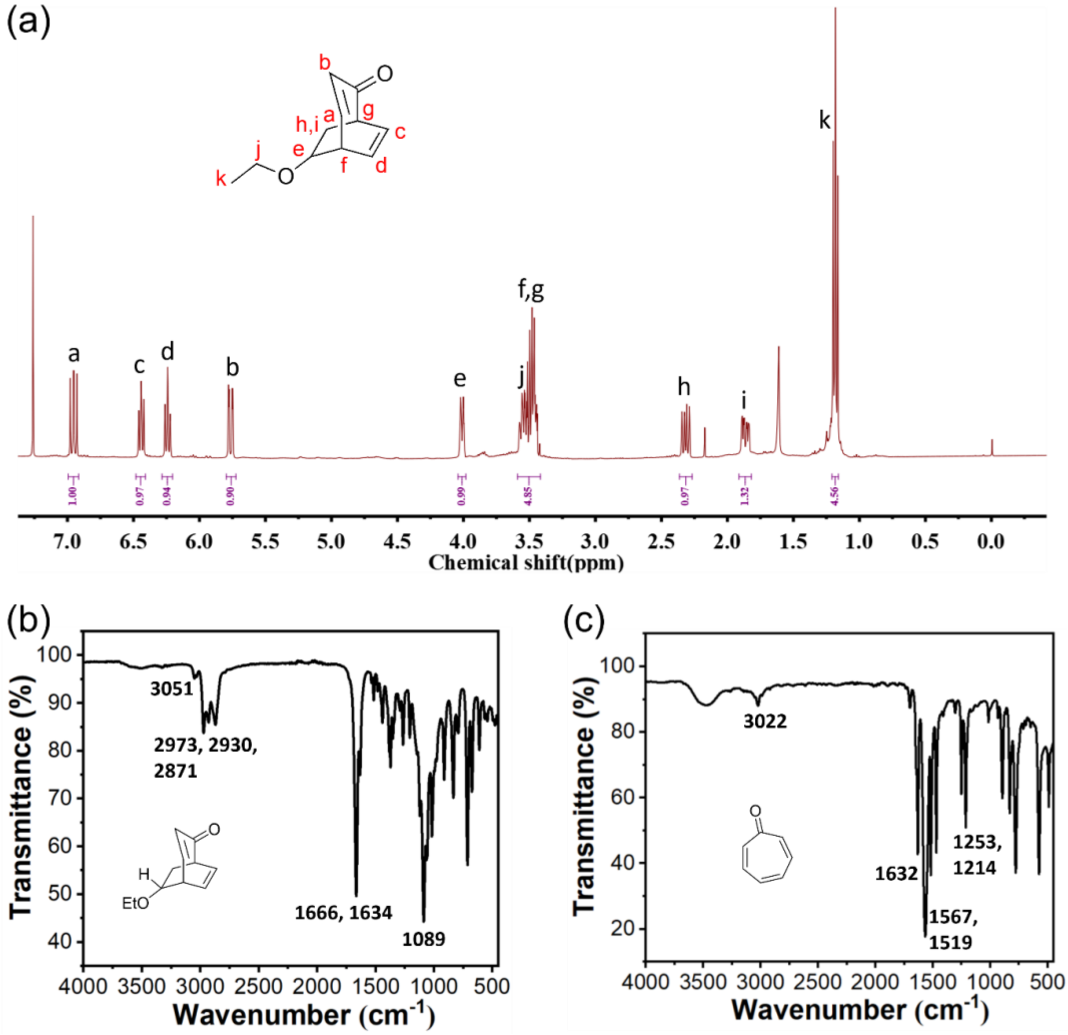

Figure S2. (a) ^{1}H NMR spectrum of Compound **1** in $CDCl_3$. FTIR spectra of (b) Compound **1** and (c) tropone.

5. Additional Raman Spectra of Compound 1 and Tropone+5%$B(C_6F_5)_3$ on Graphene

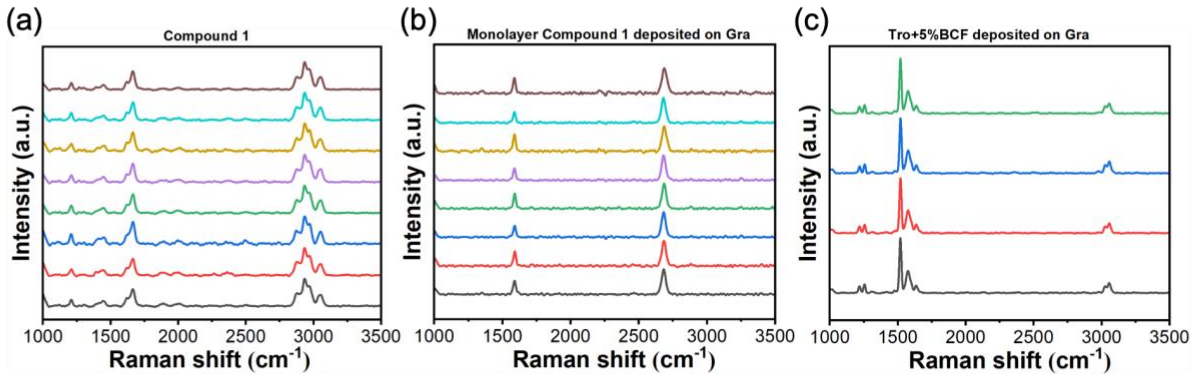

Figure S3. Additional Raman spectra of (a) compound **1**, (b) monolayer of compound **1** deposited on graphene. Sample for (b) was prepared as follows: To compound **1** (1.00 mg, 0.00561 mmol) in a centrifuge tube, 1 mL acetone was added. The solution was diluted by adding 10 μL of the solution into 1 mL of acetone to give a solution of 0.01 mg/mL. A drop (10 uL) of this solution was deposited onto graphene on 1 cm × 1 cm silicon wafer. After drying, the sample was characterized by Raman spectroscopy. (c) Additional Raman spectra of Tro+5% $B(C_6F_5)_3$ deposited on graphene.

6. Testing Cycloreversion of Tropone-Functionalized Graphene

The initial sample was prepared by treating Gra/Cu(111) with tropone at 50 °C for 1 h following the procedure described in the main text. The tropone-functionalized graphene was transferred to a silicon wafer and Raman spectra were collected (entry 1). The sample was then placed in toluene at 100 °C for 4.5 h or 19 h, or in 1,2-dichlorobenzene at 160 °C for 19 h, washed in acetone three times and dried before Raman characterization (entries 2–4). Control samples were treated in the same manner by soaking unfunctionalized graphene in toluene at 100 °C for 19 h, washing with acetone three times, and drying.

(a)

Entry	Conditions	A_{1468}/A_G	A_{1501}/A_G	A_{1535}/A_G	A_{1620}/A_G	A_{2D}/A_G
1	Initial sample	0.029 ± 0.023	0.20 ± 0.037	0.27 ± 0.071	0.011 ± 0.011	0.69 ± 0.18
2	In toluene, 100 °C, 19 h	0.0090 ± 0.012	0.29 ± 0.072	0.44 ± 0.085	0.061 ± 0.027	0.73 ± 0.31
3	In toluene, 100 °C, 4.5 h	0.0090 ± 0.018	0.17 ± 0.12	0.16 ± 0.21	0.022 ± 0.042	1.2 ± 0.53
4	In 1,2-dicholorobezene, 160 °C, 19 h	0.018 ± 0.023	0.15 ± 0.12	0.26 ± 0.22	0.042 ± 0.037	0.99 ± 0.52

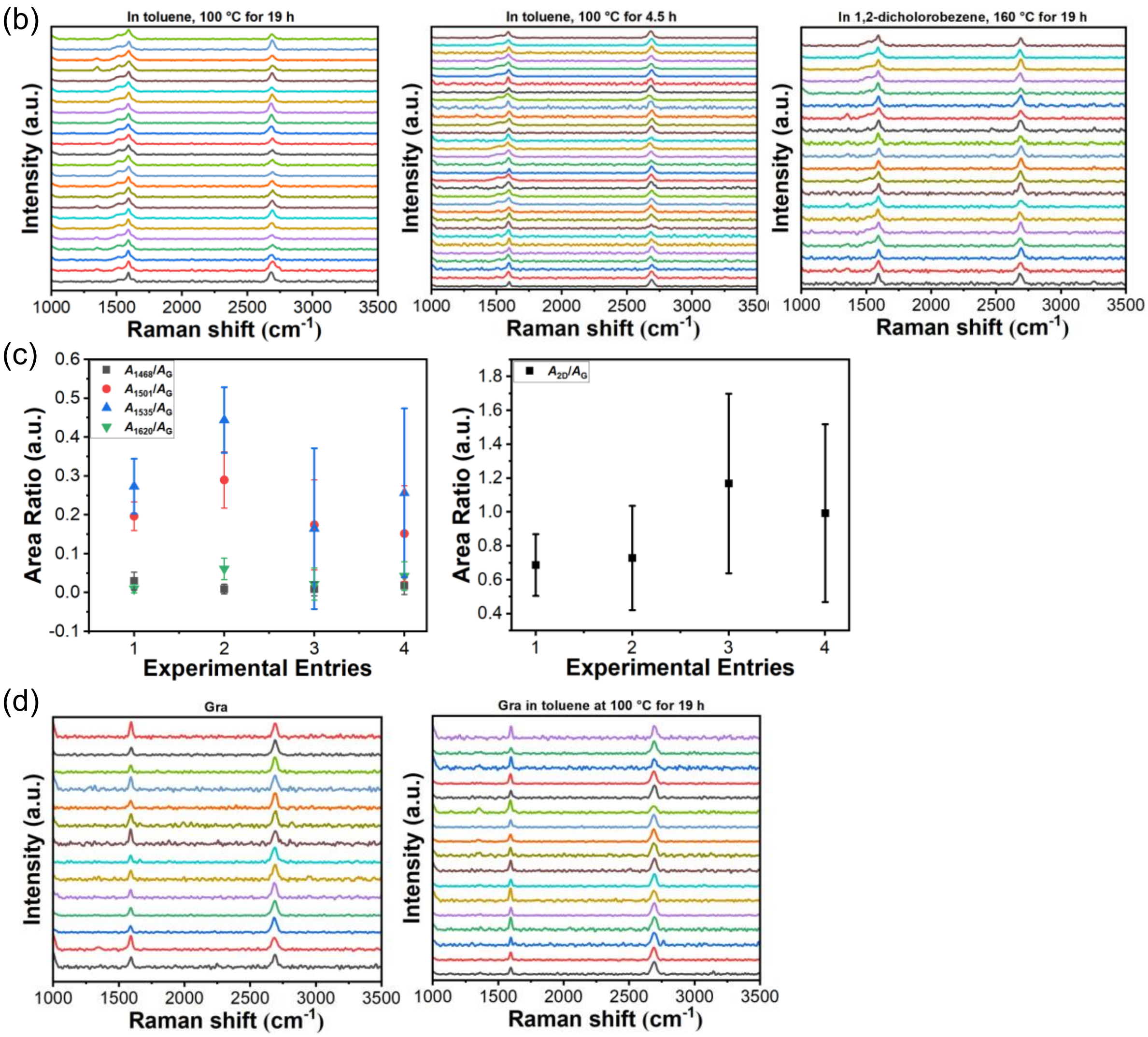

Figure S4. Testing cycloreversion of tropone-functionalized graphene. (a) Experimental conditions and summary of Raman results. (b) Raman spectra of entries 2–4. The spectra were collected at different locations on each sample, and the results are the average of all data points. (c) A_{1468}/A_G, A_{1501}/A_G, A_{1535}/A_G and A_{1620}/A_G, and A_{2D}/A_G for entries 1–4. (d) Raman spectra of control samples: unfunctionalized graphene

before (left) and after soaking in toluene at 100 °C for 19 h (right). The spectra were collected at different locations on each sample.

7. Effect of Temperature and Time on the Reaction of Gra/Cu(111) with Tropone Catalyzed by $B(C_6F_5)_3$: All Raman Spectra and Data Analysis of Samples in Figure 3

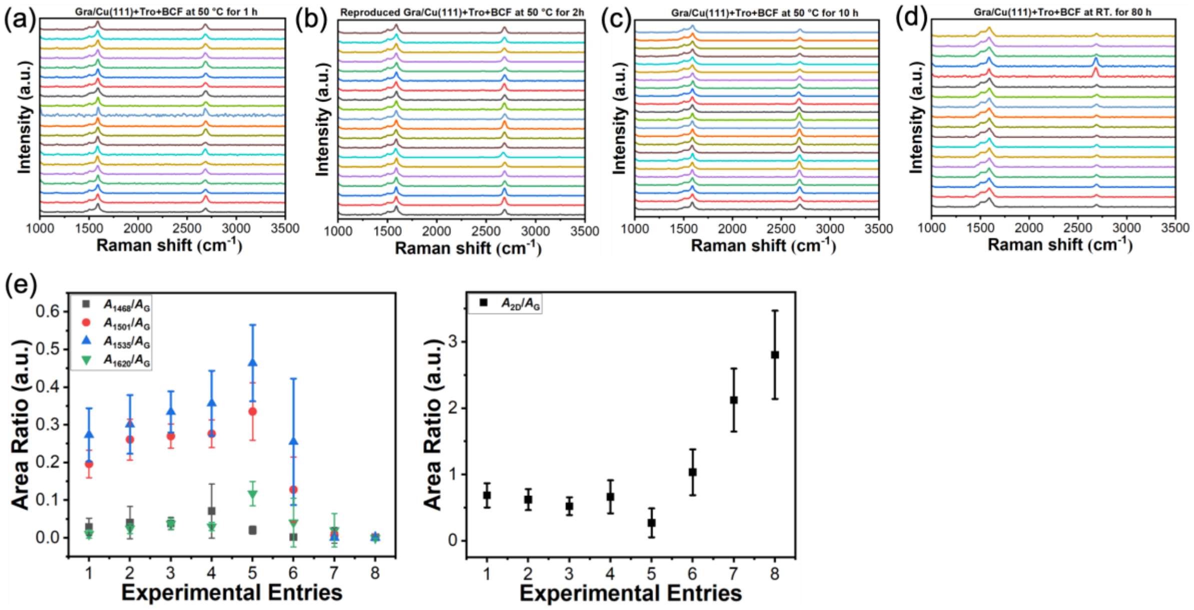

Figure S5. (a-d) Raman spectra of samples in Figure 3. Spectra were collected at random locations on two independent samples for a-c, and one sample for d. (e) A_{1468}/A_G, A_{1501}/A_G, A_{1535}/A_G and A_{1620}/A_G, and A_{2D}/A_G of all entries. Results are the average of all spectra in (a).

8. Zoomed-in XPS Spectra of Tropone-Functionalized Graphene in the Regions of B and F.

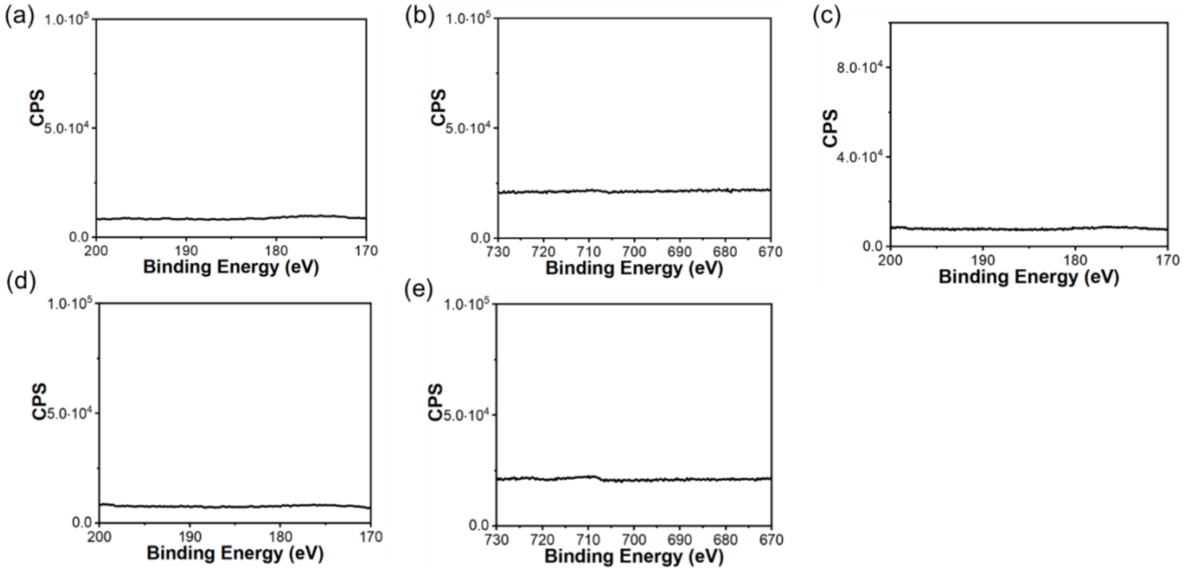

Figure S6. XPS spectra of Gra/Cu(111) after reaction with tropone (a,b) catalyzed by $B(C_6F_5)_3$ in the range of (a) 170–200 eV, (b) 670–730 eV, (c) catalyzed by $B(C_6H_5)_3$ in the range of 170–200 eV, (d,e) with 2-chlorotropone catalyzed by $B(C_6F_5)_3$ in the range of (a) 170–200 eV, (b) 670–730 eV. Neither B 1s peak at ~188.5 eV nor F 1s peak at ~689.4 eV was observed in these samples.

Tropone- or 2-Chlorotropone-Functionalized Graphene: All Raman Spectra and Data Analysis of Entries 2–4 in Figure 4

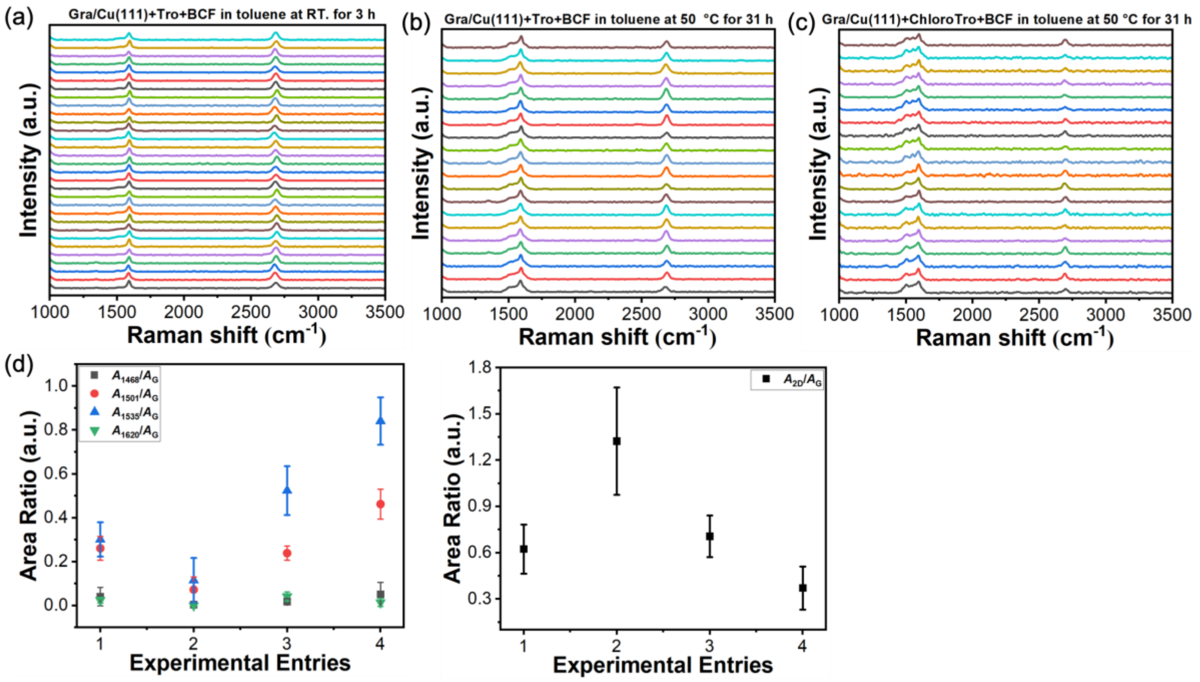

Figure S7. (a-c) All Raman spectra of entries 2–4 in Figure 4. Reactions were carried out in toluene catalyzed by $B(C_6F_5)_3$. Spectra were collected at random locations on three independent samples for (a), and two independent samples for (b) and (c). (d) A_{1468}/A_G, A_{1501}/A_G, A_{1535}/A_G and A_{1620}/A_G, and A_{2D}/A_G of all entries. Results are the average of all data points.

9. All Raman Spectra and Data Analysis of Entries 2–3 in Figure 5

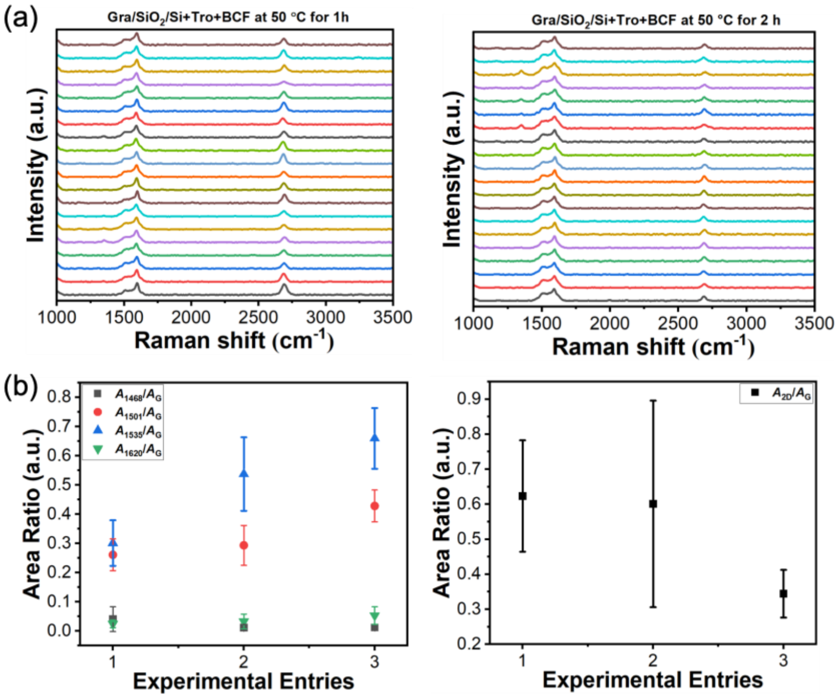

Figure S8. (a) All Raman spectra of entries 2–3 in Figure 5. Reaction was carried out by treating graphene supported on silicon wafer with tropone using $B(C_6F_5)_3$ as the catalyst. Spectra were collected at random locations on two independent samples. (b) A_{1468}/A_G, A_{1501}/A_G, A_{1535}/A_G and A_{1620}/A_G, and A_{2D}/A_G for samples in (a). Results are the average of all data points.

10. Reaction of Tropone with Gra/Cu(111) Catalyzed by BPh_3: All Raman Spectra and Data Analysis of Entry 2 in Figure 6

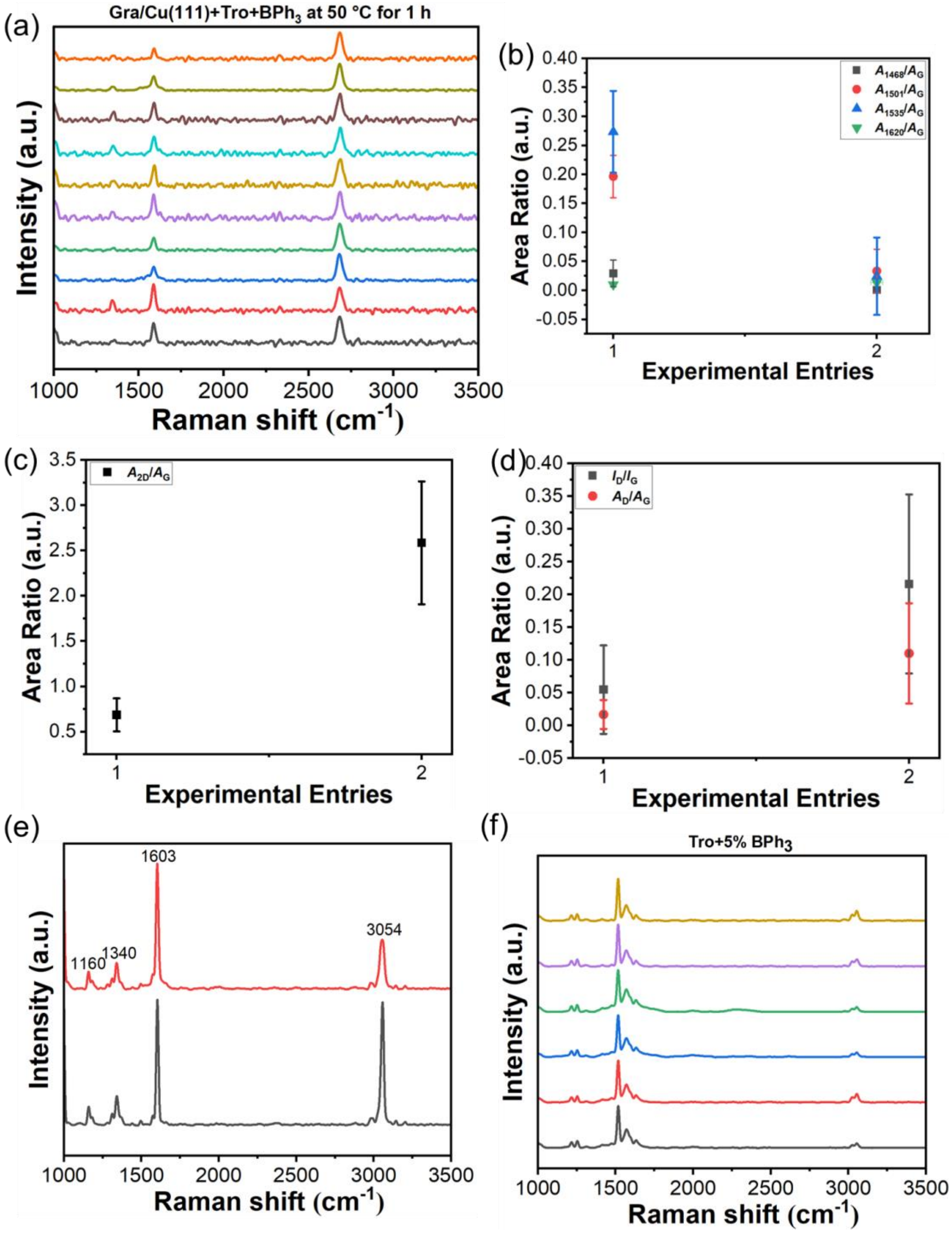

Figure S9. All Raman spectra and data analysis of entry 2 in Figure 6. Reaction was carried out by treating Gra/Cu(111) with tropone using BPh_3 as the catalyst. (a) Raman spectra, (b) A_{1468}/A_G, A_{1501}/A_G, A_{1535}/A_G and A_{1620}/A_G, (c) A_{2D}/A_G, and (d) I_D/I_G and A_D/A_G of entry 2 in Figure 6. Spectra were collected at random locations on two independent samples. Results in (b-d) are the average of all data points. Raman spectra of (e) BPh_3 and (f) Tro+5% BPh_3.

11. Calculation of L_D and n_D

The distance between two point defects, L_D, and defect density n_D, defined as the number of point defects per cm^2, were calculated according to the methods developed by Cançado et al. for Ar^+-treated graphene with a low defect density (L_D>10 nm):[12]

$L_D^2 = (1.8\pm0.5)\times10^{-9}\,\lambda^4\,(I_D/I_G)^{-1}$ (Eq. 1),
where λ (in nm) is the excitation wavelength of the laser, which was 532 nm in this study.
Since $I_D/I_G = 0.22 \pm 0.14$, $L_D = 25.7\pm8.9$ nm

$n_D = (10^7\ \text{nm})^2/\pi(L_D\ \text{nm})^2 = 10^{14}/\pi L_D^2$ (Eq. 2).
Using $L_D = 25.7\pm8.9$ nm, $n_D = (48\pm34)\times10^9\ \text{cm}^{-2}$

12. Treating Tropone-Functionalized Graphene with $NaBH_4$ or Hydrazine: All Raman Spectra, N1s XPS Spectra, and Data Analysis

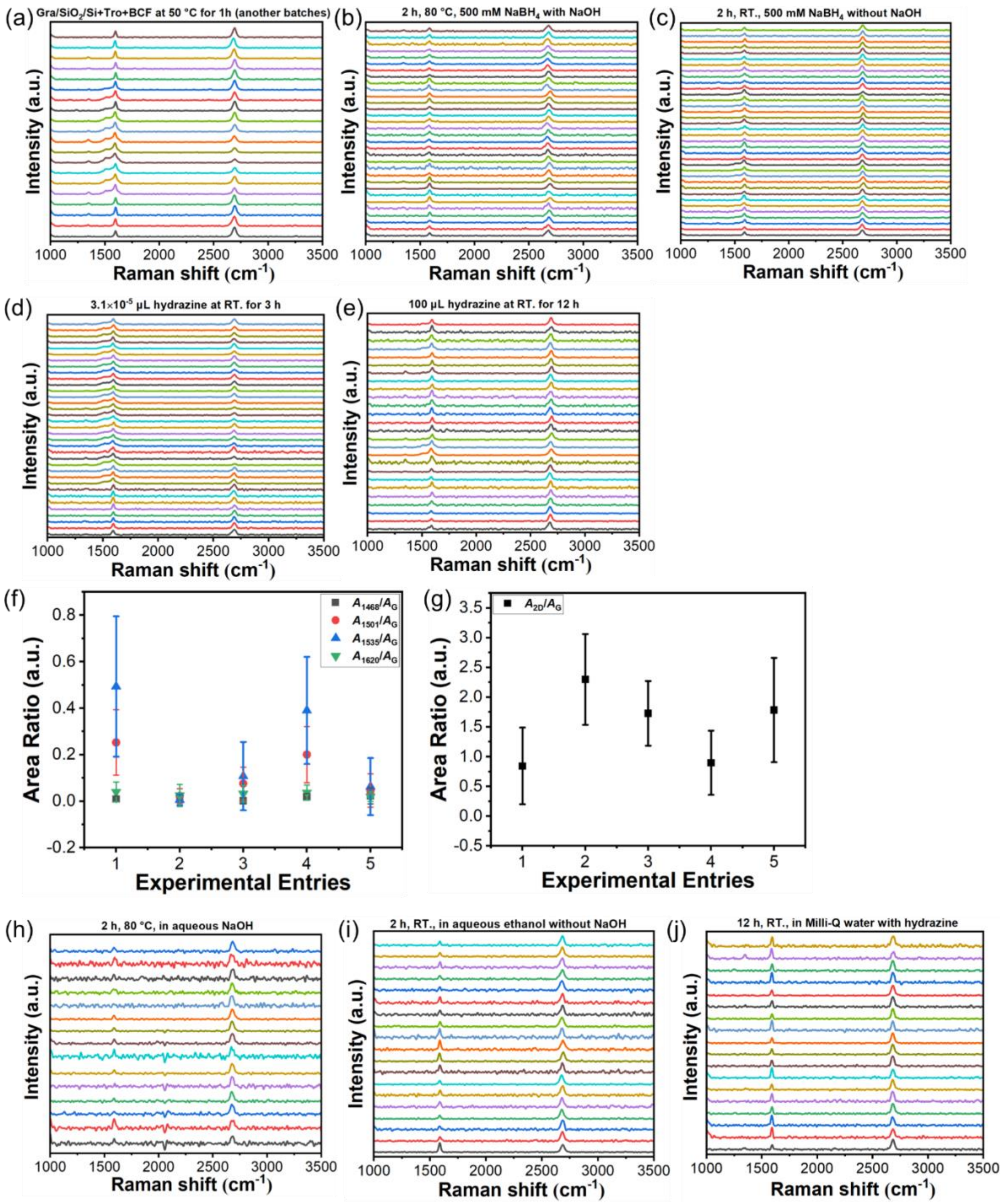

Figure S10. Raman spectra of tropone-functionalized graphene before and after reduction with $NaBH_4$ or hydrazine. (a) Initial sample of tropone-functionalized graphene supported on silicon wafer (Gra/SiO_2/Si). (b) After reaction with $NaBH_4$ in NaOH. (c) After reaction with $NaBH_4$ in 50% ethanol. (d) After reaction with 0.13 μM hydrazine. (e) After reaction with 0.4 M hydrazine. (f) A_{1468}/A_G, A_{1501}/A_G, A_{1535}/A_G and A_{1620}/A_G, and (g) A_{2D}/A_G of all samples. Raman spectra of unfunctionalized graphene after treating with (h) $NaBH_4$ in NaOH, (i) $NaBH_4$ in 50% ethanol, or (j) hydrazine in Milli-Q water.

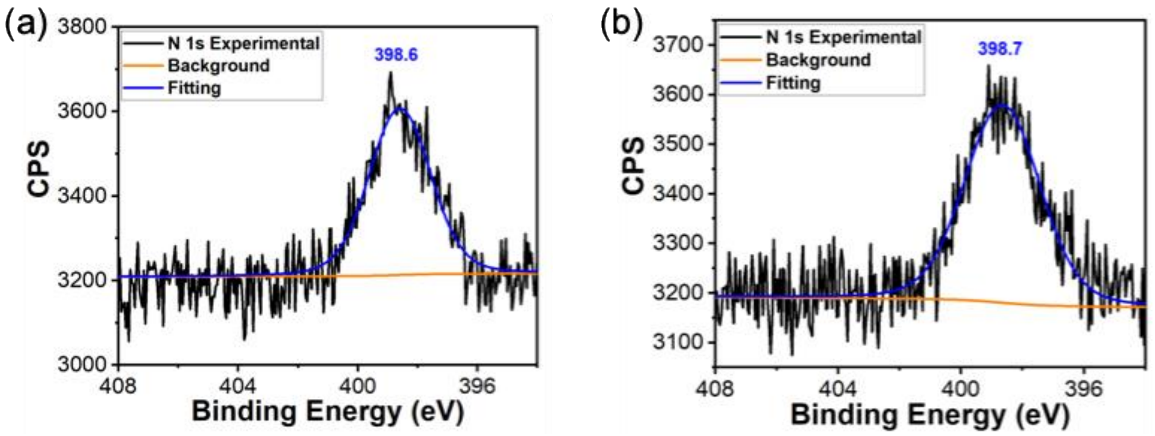

Figure S11. High-resolution XPS spectrum and deconvolution of N1s peak of tropone-functionalized graphene (a) before and (a) after treating with $NaBH_4$.

Table S4. Percentage of each C-containing functional group in tropone-functionalized graphene and after reduction with $NaBH_4$ or hydrazine, calculated from the deconvoluted high-resolution XPS C1s spectrum. The total C is normalized to 100%.

Sample	**C=C**	**C-C**	**C-O**	**C=N/C-N**	**C=O**
Tropone-functionalized graphene	61%	25%			14%
After reaction with $NaBH_4$	18%	59%	17%		5.6%
After reaction with hydrazine	24%	28%		36%	12%

13. DFT Energy Table

Table S5. SCF energies (Ry) are given for the single point energies at the 3×3×1 *k*-points on the 1×1×1 *k*-point optimized geometries.

	E (3×3×1)
tropone	-126.3112832
graphene_flat	-1218.264191
graphene_peak	-1217.702413
G/Cu	-14101.54793
trop-F	-1086.535599
$INTG_{/Cu/trop\text{-}BCF}$	-15188.15235
$INTG_{/Cu/trop}$	-14227.89974
$INTG_{/trop\text{-}BCF}$	-2304.815429
$TSG_{/trop\text{-}BCF}$	-2304.594876
$PG_{/Cu/trop\text{-}BCF}$	-15187.92297
$PG_{/Cu/trop}$	-14227.68298
$PG_{/trop\text{-}BCF}$	-2304.595638
INT	-1344.035911
TS8+2	-1343.978107
TS4+2	-1343.983159
P18+2	-1344.019808
P14+2	-1344.052561
INT-F	-2304.295471
TS-F8+2	-2304.195645
TS-F4+2	-2304.220165
P1-F8+2	-2304.229095
P1-F4+2	-2304.271133
INT-H	-1591.661565
TS-H8+2	-1591.591347
TS-H4+2	-1591.583502
P1-H8+2	-1591.626848
P1-H4+2	-1591.643091

14. Peak Graphene Constraints

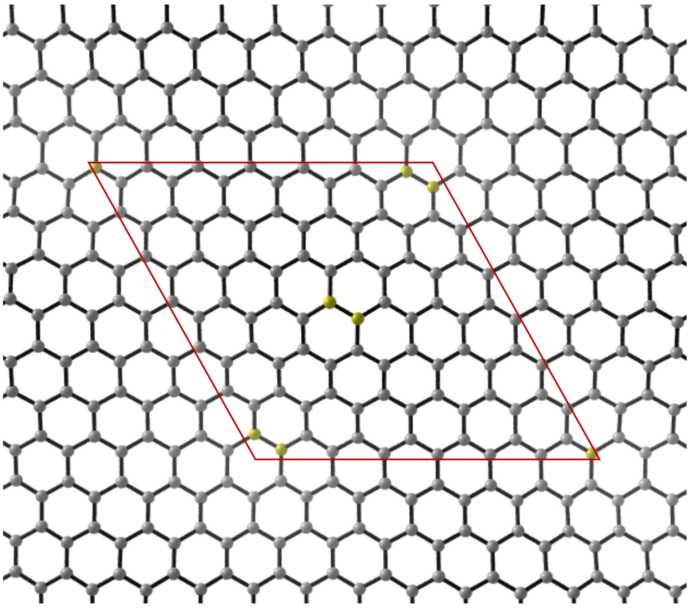

Figure S12. Peak graphene with highlighted (yellow) atoms with a fixed z-direction distance separation of 3.3 Å between the central 2 atoms and the peripheral 6 atoms on the corners of the box.

15. References

[1] S. Sarkar, E. Bekyarova, S. Niyogi, R. C. Haddon, *J Am Chem Soc* **2011**, *133*, 3324-3327.

[2] S. Bian, A. M. Scott, Y. Cao, Y. Liang, S. Osuna, K. Houk, A. B. Braunschweig, *J Am Chem Soc* **2013**, *135*, 9240-9243.

[3] S. J. Altenburg, M. Lattelais, B. Wang, M.-L. Bocquet, R. Berndt, *J Am Chem Soc* **2015**, *137*, 9452-9458.

[4] J. Li, M. Li, L.-L. Zhou, S.-Y. Lang, H.-Y. Lu, D. Wang, C.-F. Chen, L.-J. Wan, *J Am Chem Soc* **2016**, *138*, 7448-7451.

[5] L. Daukiya, C. Mattioli, D. Aubel, S. Hajjar-Garreau, F. Vonau, E. Denys, G. n. Reiter, J. Fransson, E. Perrin, M.-L. Bocquet, *ACS nano* **2017**, *11*, 627-634.

[6] M. Yu, C. Chen, Q. Liu, C. Mattioli, H. Sang, G. Shi, W. Huang, K. Shen, Z. Li, P. Ding, *Nat Chem* **2020**, *12*, 1035-1041.

[7] X. Yang, F. Chen, M. A. Kim, H. Liu, L. M. Wolf, M. Yan, *Phys Chem Chem Phys* **2022**, *24*, 20082-20093.

[8] P. A. Denis, *Chem Eur J* **2013**, *19*, 15719-15725.

[9] Y. Cao, S. Osuna, Y. Liang, R. C. Haddon, K. Houk, *J Am Chem Soc* **2013**, *135*, 17643-17649.

[10] P. Li, H. Yamamoto, *J Am Chem Soc* **2009**, *131*, 16628-16629.

[11] W. von E. Doering, F. L. Detert, *J Am Chem Soc* **1951**, *73*, 876-877.

[12] (a) L. G. Cançado, A. Jorio, E. M. Ferreira, F. Stavale, C. A. Achete, R. B. Capaz, M. d. O. Moutinho, A. Lombardo, T. Kulmala, A. C. Ferrari, *Nano lett* **2011**, *11*, 3190-3196; (b) X. Yang, F. Chen, M. A. Kim, H. Liu, L. M. Wolf, M. Yan, *Chem Eur J* **2021**, *27*, 7887-7896.
